# How Can the Desert Beetle and Biowaste Inspire Hybrid Separation Materials for Water Desalination?

**DOI:** 10.1021/acsami.0c21649

**Published:** 2021-03-01

**Authors:** Samer Al-Gharabli, Bana Al-Omari, Wojciech Kujawski, Joanna Kujawa

**Affiliations:** † Pharmaceutical and Chemical Engineering Department, German Jordanian University, Amman 11180, Jordan; ‡ Faculty of Chemistry, Nicolaus Copernicus University in Toruń, 7 Gagarina Street, Toruń 87-100, Poland

**Keywords:** polyvinylidene fluoride (PVDF), chitosan, membrane distillation, biomimetic hybrid material, molecular decoration, desalination

## Abstract

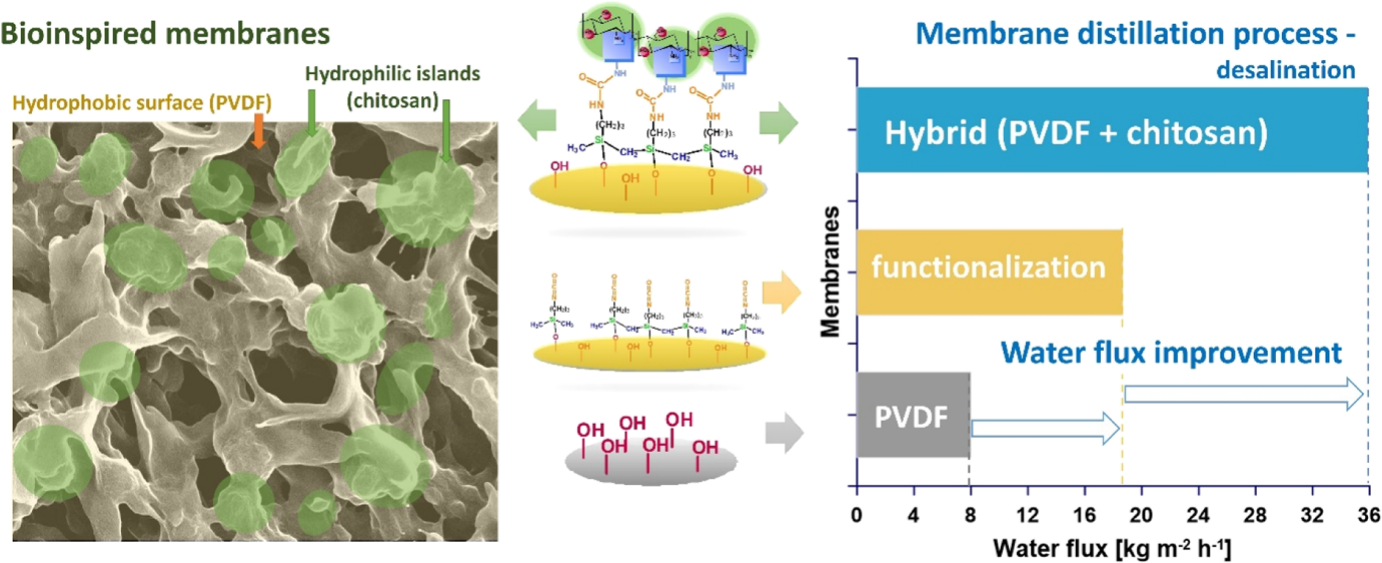

Highly effective, hybrid separation materials for water purification were generated following a bioinspired system available in nature. The desert beetle was the inspiration for the generation of separation materials. Using the hydrophobic poly(vinylidene fluoride) (PVDF) membrane as the basis, the membrane was first activated and then furnished with silane-based linkers, and the covalent anchoring of chitosan was successfully accomplished. The obtained surface architecture was a copy of the desert beetle’s armor possessing a hydrophobic matrix with hydrophilic domains. The modification was done in the presence or the lack of catalyst (*N*,*N*-diisopropylethylamine) that made it possible to tune easily wettability, roughness, and material as well as adhesive features. The membrane morphology and surface chemistry were studied by applying a series of analytical techniques. As a result of chitosan attachment, substantial improvement in transport and separation was reported. Pristine PVDF was characterized by a water flux of 5.28 kg m^–2^ h^–1^ and an activation energy of 48.16 kJ mol^–1^. The water flux and activation energy for a hybrid membrane with chitosan were equal to 15.55 kg m^–2^ h^–1^ and 33.98 kJ mol^–1^, respectively. The hybrid materials possessed enhanced stability and water resistance that were maintained after 10 cycles of membrane distillation tests.

## 1 Introduction

In recent years, water scarcity has been one of the most considerable global crises that greatly menaces the existence of organisms, including even human beings in some arid and undeveloped countries.^1,2^ Luckily, nature and natural selection have developed a variety of biomimetic inspirations.^3−6^ Water collection performances from fog have been established by different types of organisms, for example, the Namib desert beetle,^4,7,8^ cactus cluster,^6,9^ and spider silk.^3,10,11^ Particularly, the Namib desert beetle presents an interesting approach by virtue of alternating nonwaxy hydrophilic and wax-coated hydrophobic regions, exhibiting a remarkable water collection ability by capturing and coalescing drops of water on the hydrophilic areas while making them roll-off with the assistance of its hydrophobic regions. Such a solution, inspired by nature, can be adapted to the materials science for generating advanced materials for water harvesting. To do this, a hydrophilic node/island-like structure can be generated on the surface with hydrophobic or superhydrophobic features.

Many complicated and time-consuming processes requiring special instruments were presented for the construction of hierarchical patterns of superhydrophilic/superhydrophobic materials. For instance, Moazzam et al.^12^ applied a negative photolithography technique to fabricate porous surfaces with divergent wettabilities. Zahner and co-workers^13^ generated a superhydrophilic micropattern on a (super)hydrophobic thin porous polymer film by implementing ultraviolet-initiated surface photografting with a defined photomask. Yang et al. developed an electrochemical etching method to construct the superhydrophobic–superhydrophilic patterned material on superhydrophobic metal substrates. One of the interesting options for producing such hybrid materials is to attach the island-like structure made from chitosan to the polymeric materials.

Chitosan is of substantial interest for membrane modification owing to its low cost, biocompatibility, hydrophilicity, and antibacterial and antifouling features.^14,15^ Furthermore, chitosan, a natural glucosamine polymer, is classified as a biowaste. For the latter reason, to find a way of reusing chitosan is really important from the environmental point of view. The addition of chitosan during material production may not only meaningfully facilitate the material properties but also enhance the consistency and stability of the coating layer that, in the final step, will improve transport and separation across such materials. The utility of chitosan blends with a polymeric matrix, that is, fluoropolymers, was presented in the scientific literature.^16^ However, the disadvantage of losing chitosan and material inconsistency has been observed due to performance only physical modification with chitosan either by blending or coating without chemical stability.

Wang and co-workers^17^ presented the application of chitosan-poly (vinyl alcohol)/polyvinylidene fluoride (PVDF) hollow fiber composite membranes for isopropanol dehydration *via* pervaporation. Although the results were interesting, the attachment of chitosan was accomplished only by the physical process of immersion. A good separation performance has been found for the membrane with 60 wt % chitosan and 0.1 wt % glutaraldehyde. The membrane was characterized by a separation factor equal to 2140 and a permeate flux equal to 306 g m^–2^ h^–1^. The measurements were performed at 60 °C, and the feed solution contained 90% isopropanol. When the water content changed from 3 to 15 wt %, the permeate flux enlarged from 207 to 346 g m^–2^·h^–1^, while the separation factor decreased from 2406 to 1876.

The composite membranes containing PVDF and chitosan-coated layers were produced and tested by the Jiraratananon’s group.^18,19^ One of the works showed the application of composite membranes for protection against wetting by oils from fruit juice and for reduction of flavor losses (e.g., limonene) in the osmotic distillation process. It was observed that the coated membranes not only presented higher water flux but also gave lower flavor flux. The established results suggested that the generated coated membranes were appropriate for a feed containing high limonene oil (500 ppm).^18^ The second example was focused on the fouling limitation owing to the hydrophilicity increase by chitosan coating from a contact angle (CA) equal to ca 115° for pristine PVDF to 61.5° for a PVDF-chitosan (1 wt %) composite prepared by a combined flow through and surface flow methods.^19^ However, the lack of a suitable method for generating a stable, covalently bonded layer of chitosan to a polymeric matrix was a great motivation to develop such a solution.

A two-step procedure, with furnishing of a polymeric surface with a silane-based modifier and then with chitosan, was established. The functional silane modifiers containing organofunctional or organoreactive moieties can be applied to conjugate biomolecules to inorganic substrates, for example, polymeric or ceramic ones.^20^ The suitable choice of the reactive or functional groups for a specific usage can make possible the attachment of a wide variety of molecules, for example, oligonucleotides, proteins, whole cells, or even tissue sections to substrates. The organosilanes applied for the abovementioned utilizations comprise reactive or functional groups such as amino, epoxy, hydroxyl, aldehyde, thiol, carboxylate, and even alkyl groups to bind molecules through hydrophobic interactions. During the modification process, both reactive and terminal groups are important and can determine the effectiveness of the functionalization process.

**Figure 1:**
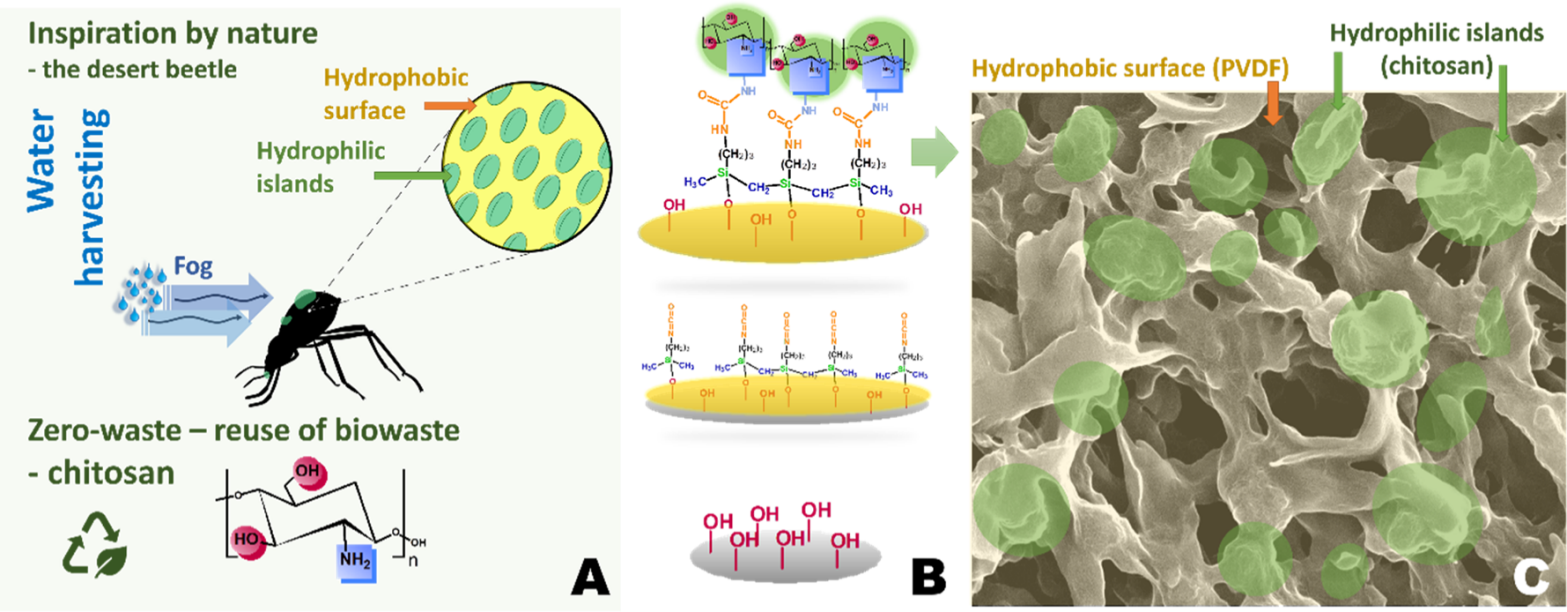
Idea of the work. (A) Specific hydrophobic surface of desert beetle with hydrophilic islands and reused biowaste material, chitosan. (B) Schematic presentation of membrane modification route. (C) Biomimicry of desert beetle surface, SEM image.

This work was intended to produce nature-inspired hybrid separation materials. Biomimicry was performed by generating chitosan hydrophilic islands by anchoring chemically to the PVDF membranes (Figure 1), which had been activated and furnished with silane linkers. Moreover, the goal was to characterize materials systematically. Finally, material features were used to understand in a better way how the modification influenced transport and separation performance. Membranes were subsequently evaluated during the desalination process.

## 2 Experimental Section

### 2.1 Materials

PVDF membranes with a nominal pore size of 0.22 μm were purchased from Merck Millipore (Germany). Methyl acetate (MeAc), methanol (MeOH), dimethylformamide (DMF), dichloromethane (DCM), toluene, hydrogen peroxide (H_2_O_2_) 30%, 3-isocyanatopropyldimethylchlorosilane (M-Cl), and *N*,*N*-diisopropylethylamine (DIPEA) were bought from ABCR Chemicals (Germany). Chitosan (deacetylation 75%, low molecular weight—mol weight 50–190 kDa) was supplied by Sigma-Aldrich (Germany). In addition, sodium chloride (NaCl) was purchased from Avantor Performance Materials (Poland). Aqueous solutions were prepared using deionized water (15 MΩ·cm) (Elix, Millipore).

### 2.2 Analytical Methods and Equipment

#### 2.2.1 Characterization of the Material

For the characterization of surface modification, attenuated total reflection (ATR) Fourier transform infrared (FTIR) technique was applied for the characterization of surface modification effectiveness using Bruker VERTEX 80v. 512 scans were collected with a resolution of 4 cm^–1^.

The morphological properties of the membranes were characterized by scanning electron microscopy (SEM) (Quantax 200) with an XFlash 4010 detector (Bruker AXS machine, Czech Republic) and with a secondary electron (SE) detector. For samples, imaging sputtering with a gold nanolayer (Au thickness layer—5nm) was implemented to enhance the sample’s conductivity.

Pore size distribution and the pore size of the material were determined based on the modified bubble point method using Coulter Porometer II (Coulter Electronics Ltd., UK).^21^ Samples (2.5 cm diameter) were immersed in Porefil wetting liquid (γ_L_ = 16 mN m^–1^) before measurements. Three samples were taken from each membrane to determine an average value.

Roughness parameters (*R*_a_) were determined with a Veeco (Digital Instrument, England) atomic force microscope equipped with NanoScope IIIa Quadrex (England) based on an integrated mathematical algorithm in NanoScope Analysis Software (1.40, Build R3Sr5.96909, 2013 Bruker Corporation). The mean roughness (*R*_a_) is the arithmetic average of the absolute values of the roughness profile ordinates. *R*_a_ is the most efficient and precise surface roughness measure frequently implemented in engineering and materials science. *R*_a_ is sensitive on the small differentiations in the roughness and describes very well the height variations of the surface. The dynamic mode was selected, and the silicone nitrate probe (SNL-10, Bruker, spring constant 0.48 N m^–1^) was used. All analyses were done in triplicate at room temperature with a scanning area equal to 5 × 5 μm. Adhesion force (*F*_a_), Young modulus (*E*), and nanohardness (*H*) parameters were selected for the mechanical characterization and were accomplished at contact option applying diamond tip (*k*, spring constant 225 N m^–1^, PDNISP, Bruker). The detailed procedure was described elsewhere.^15,22^

The wettability study, including determination of CA, surface free energy (SFE), and critical surface (γ_cr_) tension, was done with a goniometer (Attention Theta from Biolin Scientific, Gothenburg, Sweden). Water, diiodomethane, glycerol, *N*,*N*-dimethylformamide, xylene, toluene, *n*-dodecane, cyclohexane, hexane, and mixers used as feed solutions were utilized as testing liquids during the goniometric measurements with a constant volume of 3 μL and 5 s equilibration time. CA was determined with ±0.5° accuracy at room temperature. SFE was calculated based on the Owens, Wendt, Rabel, and Kaelble method.^23^ The surface tension of testing liquids during separation was determined according to the pendant drop method and Laplace–Young equation at room temperature and temperature of the separation process.^24^ This new approach was designed to understand more precisely how the membrane material behaves during the separation. Liquid entry pressure (LEP) was another parameter used for the wettability study of the prepared materials. LEP for water was established according to the Cantor–Laplace equation (eq 1).^15,25,26^
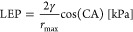
1where γ is the surface tension of testing liquid (0.07199 N m^–1^ for water at 25 °C), cos(CA) is the cosine of the CA generating by water on the membrane surface, and *r*_max_ is the maximum pore radius for the membrane (bubble point).

The XRD—X-ray diffraction method was used for the determination of phase composition. Spectra were collected in the angles range (2θ) between 5 and 90°. The step and rate were fixed on 0.02° and 3°/min, respectively. The analyses were accomplished at room temperature. A Philips X’Pert PW 3040/60 diffractometer (Kα = 1.5418 Å) with a Cu lamp (30 mA and 40 kV) was used. For data analysis, Kα2/Kα1 correction and Rachinger’s method were employed.^15,25,26^

### 2.3 Modification of Membranes

#### 2.3.1 Activation Process

PVDF-rounded samples with 47 mm diameter were activated according to the established method with a diluted (i.e., 20 wt %) solution of piranha activator (30 min treatment at 60 °C).^15,27^ Membranes equipped with hydroxyl groups (OH) were produced during the activation step. In brief, the PVDF membrane was soaked in MeOH, subsequently placed in glass bottles (50 mL), and finally 10 mL of piranha solution was added. The membrane and activator were set and mixed for 30 min at 60 °C. The activated membranes were labeled P-OH. To quench the reaction, the membranes were immersed in a water bath for 5 min and then cleaned with methanol and water five times in each solvent. In the final step, the membranes were dried at 70 °C (12 h).

#### 2.3.2 Membrane Silanization

Activated, dried membranes (P-OH) were modified with a silane-based modifier, that is, 3-isocyanatopropyldimethoxychlorosilane (M-Cl). The applied solvent, that is, DCM, was stored over molecular sieves to assure the absence of water in the solvent. The sample was placed in the 0.1 M DCM solution of the grafting agent (M-Cl) for 3 h at 35 °C. The modification process was accomplished under an ambient atmosphere of nitrogen in the glovebox. The membranes were subsequently washed in DCM, methanol, and water and dried for 12 h at 70 °C. The purpose of the reaction was to generate a stable covalent connection between reactive groups of modifiers (chlorine group) and hydroxyl groups available on the activated PVDF. The isocyanate groups of grafting agents remained unused and accessible to the next step of the modification. The produce samples were labeled in the following way P-M-Cl.

To increase the effectiveness of the silanization process, DIPEA was added as a catalyst. DIPEA was added to the P-OH sample, which was placed in a glass bottle with 0.1 M DCM solution of M-Cl in the molar ratio 1:1 (M-Cl/DIPEA). The purpose of DIPEA addition was to absorb the generated byproduct (HCl) and to increase the efficiency of the silanization reaction. Grafting was performed at the same condition of time (3 h) and temperature (35 °C). The generated membrane was labeled P-M-Cl-DIPEA. The catalyst addition was aimed to increase the efficiency of the grafting process.

#### 2.3.3 Generation of Hybrid Membranes

The chitosan (0.1 g) was mixed/dispersed in 3 mL of DCM (stored with molecular sieves) for 4 h at 35 °C. The silanized membrane was placed in a glass bottle with well-mixed chitosan, and the mixing was continued for 24 h at 35 °C. In the next step, the sample was cleaned in the following solvents, DCM, and distilled water. The cleaning procedure was repeated five times for each solvent in the sonication bath for 5 min. The goal of the purification step was to remove all chitosan particles that were not covalently attached to the silanized materials. The membranes were dried in an oven for 12 h at 40 °C. The produced hybrid samples were assigned in the following way: P-M-Cl + CS and P-M-Cl-DIPEA + CS.

### 2.4 Membrane Characterization in Desalination

The experiment intended to assess the utility of the generated hybrid membranes in desalination process. The AGMD—air-gap membrane distillation experimental equipment has been shown elsewhere.^15,28^ An important part was to calculate apparent activation energy (*E*_a_) for water transport across all samples. For that purpose, diverse driving forces (i.e., 85, 120, and 220 mbar ±0.68 mbar) created by various temperatures of feed (46, 52, and 65 ± 2 °C) were selected.^15,25,26,28^ The driving forces were determined based on Bulk’s equation (eq 2) and established by using different feed temperatures and constant permeate temperature (8 ± 2 °C).^29^
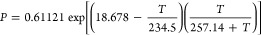
2where *T* is the temperature in [°C] and *P* is the pressure in [kPa].

To study the activation energy of the transport of water throughout the membrane,^30^ the temperature-dependent phenomenon developed by Eyring et al.^31^ was implemented. The relation between transport, permeate flux, and temperatures is generally described by the Arrhenius-type equation (eq 3).^32^ By implementing the above presented idea, it allows to investigate deeply the relation between temperature and permeate flux.^15,28^
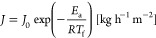
3where *J*_0_ is the pre-exponential factor (i.e., the flux of the permeate at infinite temperature), *E*_a_ is the apparent activation energy for flux of the permeate, *R* is the gas constant, and *T*_f_ is the temperature of feed solution.

To understand in a better way how the modification process, particularly the formation of hybrid membranes, influences the transport across the membranes, the overall mass transfer coefficient (*K*) and the permeability of the generated separation materials were calculated. To determine the permeance coefficient (*P*_i_/*l*) for water transport, the Baker’s approach has been implemented.^33^ The *K* parameter (eqs 4 and 5) depends on the properties of the membrane material, for example, morphology, porosity, tortuosity, thickness, and pore size,^34^ and was calculated based on the presented equations
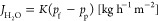
4
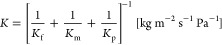
5where *K* is the overall mass transfer coefficient (kg m^–2^ s^–1^ Pa^–1^), *p*_f_ is the partial vapor pressure of water in the feed, *p*_p_ is the partial vapor pressure of water in the permeate, *K*_f_ is the mass transfer coefficient of the feed layer, *K*_m_ is the mass transfer coefficient of membrane, and *K*_p_ is the mass transfer coefficient of the permeate layer.

Finally, after careful study of the transport properties, the separation was assessed as well. The transport separation properties were studied using water and 0.5 M NaCl as feed solutions. The separation features were assessed based on NaCl rejection coefficient (*R*_NaCl_) (eq 6) during desalination *via* membrane distillation. The salt concentration was measured with a conductometer (Elmetron CPC-505 conductometer, Poland).
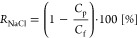
6where *C*_p_ is the salt content in permeate and *C*_f_ is the salt content in the feed.

## 3 Results and Discussion

### 3.1 Material Activation—Spectroscopic Characterization

The accomplished research offers several distinct advantages, including an easy and effective method for the production of hybrid separation materials with tunable features depending on the selected route of treatment. In the beginning, the generation of the desired, hybrid materials was confirmed by implementing a number of analytical methods. In the first step, the PVDF inorganic support was activated based on the developed and systematically characterized method.^22^ During the process, the materials rich in hydroxyl groups were formed. The high efficiency of the reaction has been confirmed by an ATR–FTIR technique. The gained spectra revealed the formation of hydroxyl groups (OH) owing to the presence of a wide band for hydroxyl groups (OH) at 3333 cm^–1^ (Figure S1).^15,27^ Furthermore, new bands were observed at 3320, 2972, 2931, and 2876 cm^–1^ on the spectrum of hydroxylated support (P-OH). The bands were associated with the activation process and with proving the fluorine reduction in the PVDF matrix, followed by the substitution of −CF with −CH.^15,22,27^

### 3.2 Silane-Based Linker Attachment—Spectroscopic Characterization

**Figure 2:**
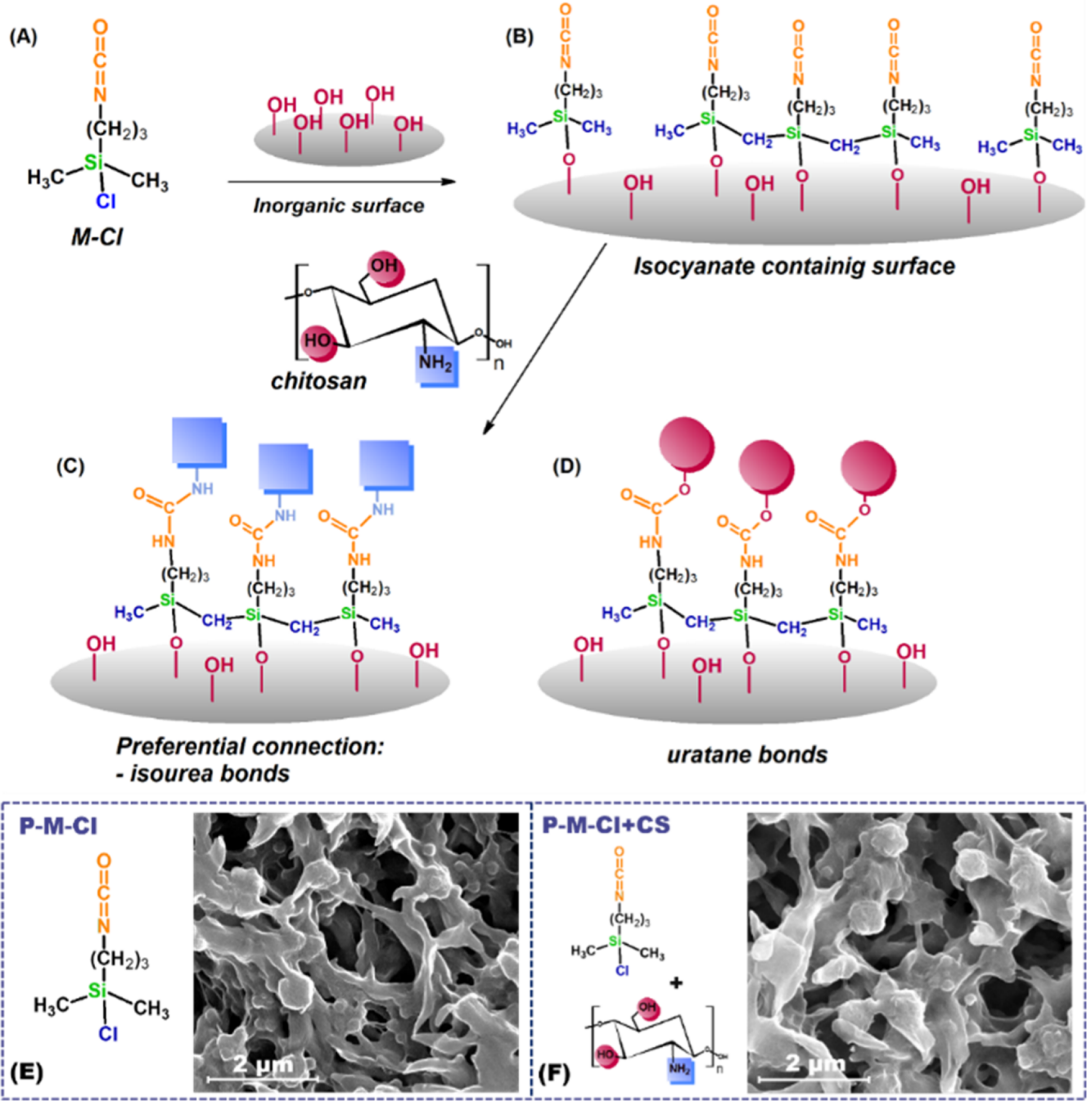
Scheme of hybrid material generation. Example with M-Cl modifier (A). Effect of material silanization (B). Hybrid materials—connection of chitosan by the amine group (preferential connection) (C) and by the hydroxyl groups (D). SEM images of the generated materials (E,F).

Samples rich in hydroxyl groups were subsequently used during the grafting process with isocyanate-terminated organosilanes, that is, 3-isocyanatopropyldimethylchlorosilane (M-Cl) (Figure 2). The modifiers offer high reactivity owing to the isocyanate group, and on the other hand, the molecule beads the classical behavior of dialkyl-monochlorosilanes.^35^ This dual-phase characteristic made them very helpful spacers. A significant advantage was to select spacers with chlorine groups instead of the ethoxy ones owing to the reactivity and possibility for tuning the surface characteristic of the generated materials.^20^ Depending on the requirements and way of modification, the monochloro modifier can be either deposited on an inorganic substrate using high purity and dry organic solvent to promote hydrolysis of the alkoxy groups before coupling or can be attached covalently to the substrate with a layer of silane compounds.^20^ In the latter case, the advantage is the prospect to generate a thinner, more controlled layer of the silanes that was selected for the medication with M-Cl. This method can produce a monolayer of functional propyl groups on the surface, for example, amino, glycidoxy, carboxyethylo, or isocyanate.^20^ The 3-isocyanatopropyldimethylchlorosilane (M-Cl) was carefully selected to the covalent attachment of chitosan possessing the both, amine and hydroxyl groups (Figure 2). The isocyanate terminal group is extremely reactive and particularly valuable for covalent coupling to the amine or hydroxyl groups under nonaqueous conditions. An isocyanate reacts with amines forming isourea (−NHCONH) linkages and with OH groups forming carbamate (urethane) bonds (−NHCOO−).^36^ Both reactions can occur in an organic solvent to couple molecules to inorganic substrates. However, in the case of chitosan, the isourea connection with the utilization of amine groups should be generated preferentially owing to the much faster reaction of the NCO group with amine.^37,38^

**Figure 3:**
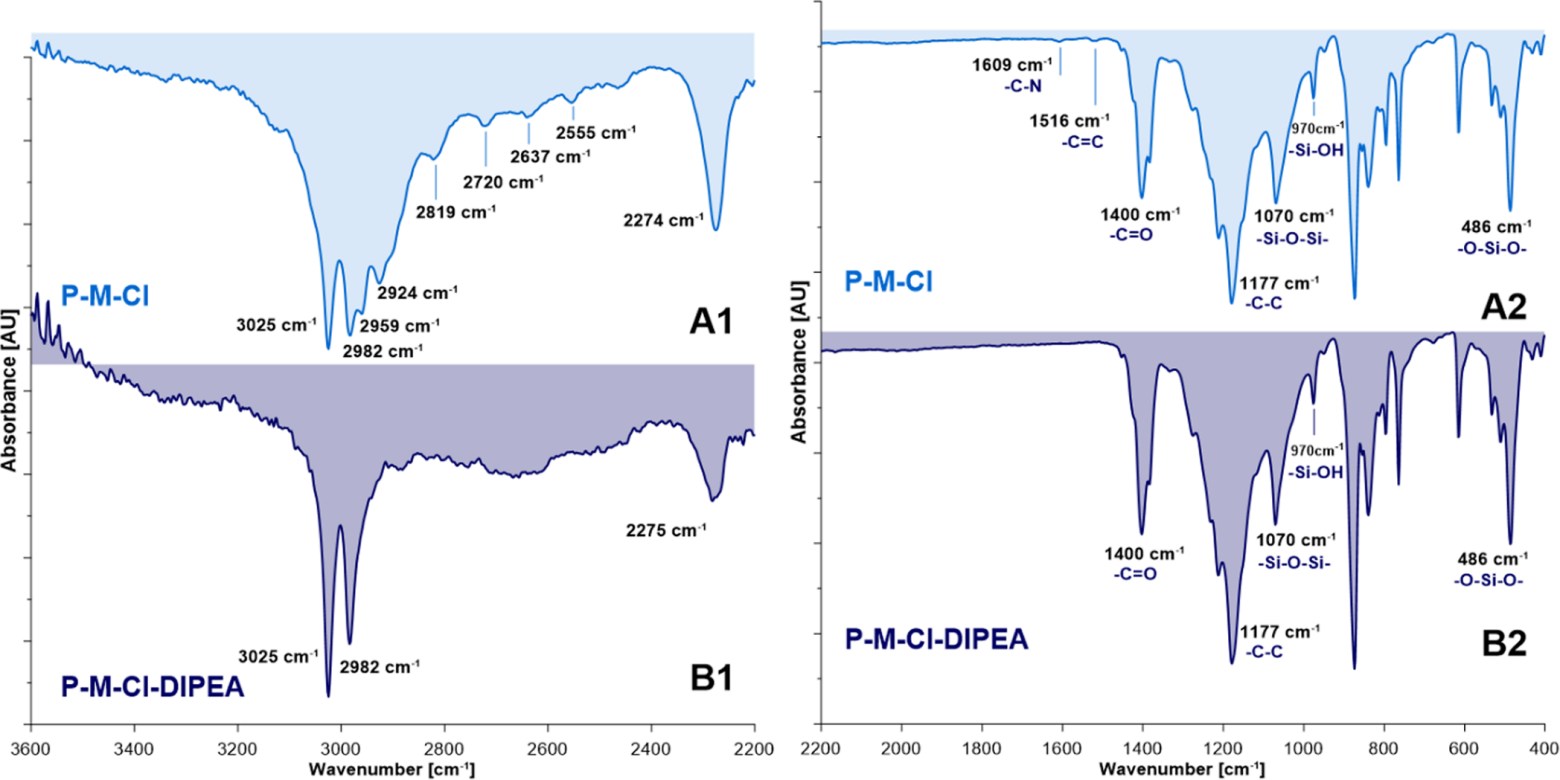
ATR–FTIR spectra of silanized membranes: (A1,A2) P-M-Cl and (B1,B2) P-M-Cl-DIPEA.

In accordance with the presented way of modification (Figure 2), the grafting agents (Figure S2) were covalently attached to the membrane surface using chlorine (M-Cl) reactive groups. The reaction took place between these groups and the hydroxyl ones available on the membrane surface leaving the isocyanate group free for further modification. This statement was proven by the presence of following bands, found for all silanized samples (Figure 3), which appeared on the spectra in the range of 3000–2840 cm^–1^ (asymmetric and symmetric −CH_2_ stretching bonds) and −C–C–C– at 1177 cm^–1^ associated with the presence of modifiers and their alkyl chain part. Moreover, the bands at 1423 and 1400 cm^–1^ were associated with the asymmetric vibration ones of CH_3_ and in-plane deformation of −CH_2_. More important was to see the bands in the range of 1100–480 cm^–1^ that proved the connection of modifiers with an inorganic support furnished with hydroxyl groups. Peaks at 1070 (−Si–O–Si−), 615 cm^–1^ (connection Si–O–substrate), and 486 cm^–1^ (−O–Si–O−) evidenced the covalent grafting of the PVDF membranes (Figure 3).^39^ The observed bands at 970 cm^–1^ possessing low intensity indicate that some hydroxyl groups were still available after the grafting process with modifiers. This finding is in good agreement with the literature, showing that during the modification process, the hydroxyl groups can remain unused mostly owing to the steric effect of modifiers and limited access to the inorganic substrate.^27,40^ It was essential to observe an intensive peak at 2274 cm^–1^ ensuring that isocyanate groups were not used and are available for further modification.

### 3.3 Hybrid Material Formation—Spectroscopic Characterization

**Figure 4:**
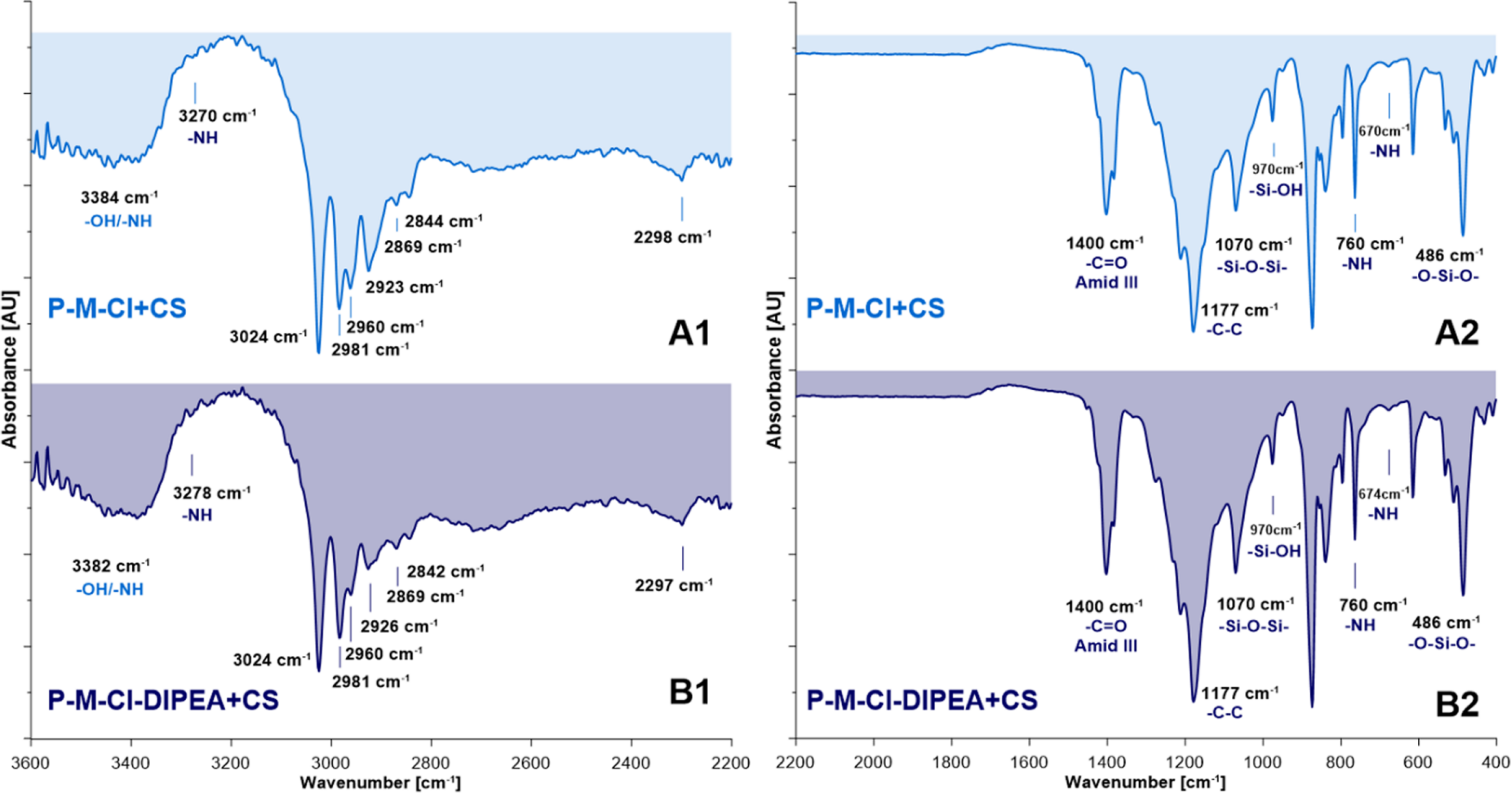
ATR–FTIR spectra of hybrid membranes: (A1,A2) P-M-Cl + CS and (B1,B2).

In the second step of modification, the CNO group was the most important element, generating a connection with chitosan molecules (Figure 4). Isocyanates can react with compounds holding active hydrogen atoms, for example, alcohols, amines, water, mercaptanes, or carboxylic acids. They can also react themselves, generating in that case dimers (uretdiones) or trimers (isocyanurates) as well as polymerized to polyisocyanates species.^41,42^ During the reaction of isocyanate, an important part is the lack or the presence of the catalyst. In the presented work, a catalyst (Figure S2) was selected to improve the effectiveness of the material modification with silanes and then with chitosan.

Generally, without a catalyst, the positively charged C of the NCO is attacked by the nucleophilic oxygen of an alcohol group while its active hydrogen is introduced to the negatively charged N (nucleophilic addition to C=N bond).^43^ The rate of addition is directly related to the nature of the nucleophile, and the reactivity in uncatalyzed NCO reactions changes in the following way: primary aliphatic amines > secondary aliphatic amines ≫ aromatic amines > primary alcohols > water > secondary alcohol ≫ carboxylic acid > ureas ≫ urethanes.^44^

The NCO reactions are really vulnerable to catalysis, that is, catalysts mainly rise the rate of nucleophilic addition of compounds with active H to the C=N bond. There are many types of suitable catalysts based on tertiary amines, for example, DIPEA, dimethylethanolamine, diazabicyclooctane, and triethylene diamine. However, the strongest one is dibutyltin dilaurate.^42,44^ The catalysts polarize either the isocyanate or the hydroxyl compound and therefore make the C=N bond more disposed to the nucleophilic addition of the hydroxyl group. In the case of tertiary amine catalysts, it is supposed that the tertiary amine and isocyanate form an activated complex that simplifies the nucleophilic addition of the alcohol to the N=C double bond.^45^ The amines catalytic activity is relatively proportional to their base strength excluding the situation when steric hindrance interferes with the generation of the intermediate state. This description explains the differences observed on the infrared spectra for the materials formed in the presence of catalyst, DIPEA (Figure 3B).

In the second step of PVDF membrane modification, that is, hybrid material formation, the chitosan was covalently anchored to the conjugated silanes by the isocyanate group. Such a connection might occur either by amine or hydroxyl groups. The isocyanate terminal group is extremely reactive and particularly valuable for covalent coupling to the amine or hydroxyl groups under nonaqueous conditions. An isocyanate reacts with amines to form isourea (−NHCONH) linkages and with hydroxyls to form carbamate (urethane) bonds (−NHCOO−).^36^ Both reactions can take place in an organic solvent to conjugate molecules to inorganic substrates. However, in the case of chitosan, the isourea connection with the utilization of amine groups should be generated preferentially owing to the much faster reaction of the NCO group with amine.^37,38^ The first sign for successful chitosan attachment was the reduction/disappearance of the characteristic band for isocyanate, indicating the reaction (Figure 2). In all cases, the significant reduction of the NCO peak at 2270 cm^–1^ was noticed (Figure 4). Subsequently, the presence of a shoulder band at 3380 cm^–1^ for P-M-Cl + CS and P-M-Cl-DIPEA + CS was related to OH/NH bands from the chitosan structure. Additionally, the spike at 3278 cm^–1^ of −NH supports the successful connection of chitosan. Although the catalyst was applied, both samples possessed comparable effectiveness in grafting and chitosan attachment. The observed broadband at 3361 cm^–1^ confirmed the high level of chitosan connected to the membrane, and the characteristic peaks for isouratene connection (1690, 1636, and 1536 cm^–1^) proved that chitosan was linked by the amine-reactive group (Figure 2C).

**Figure 5:**
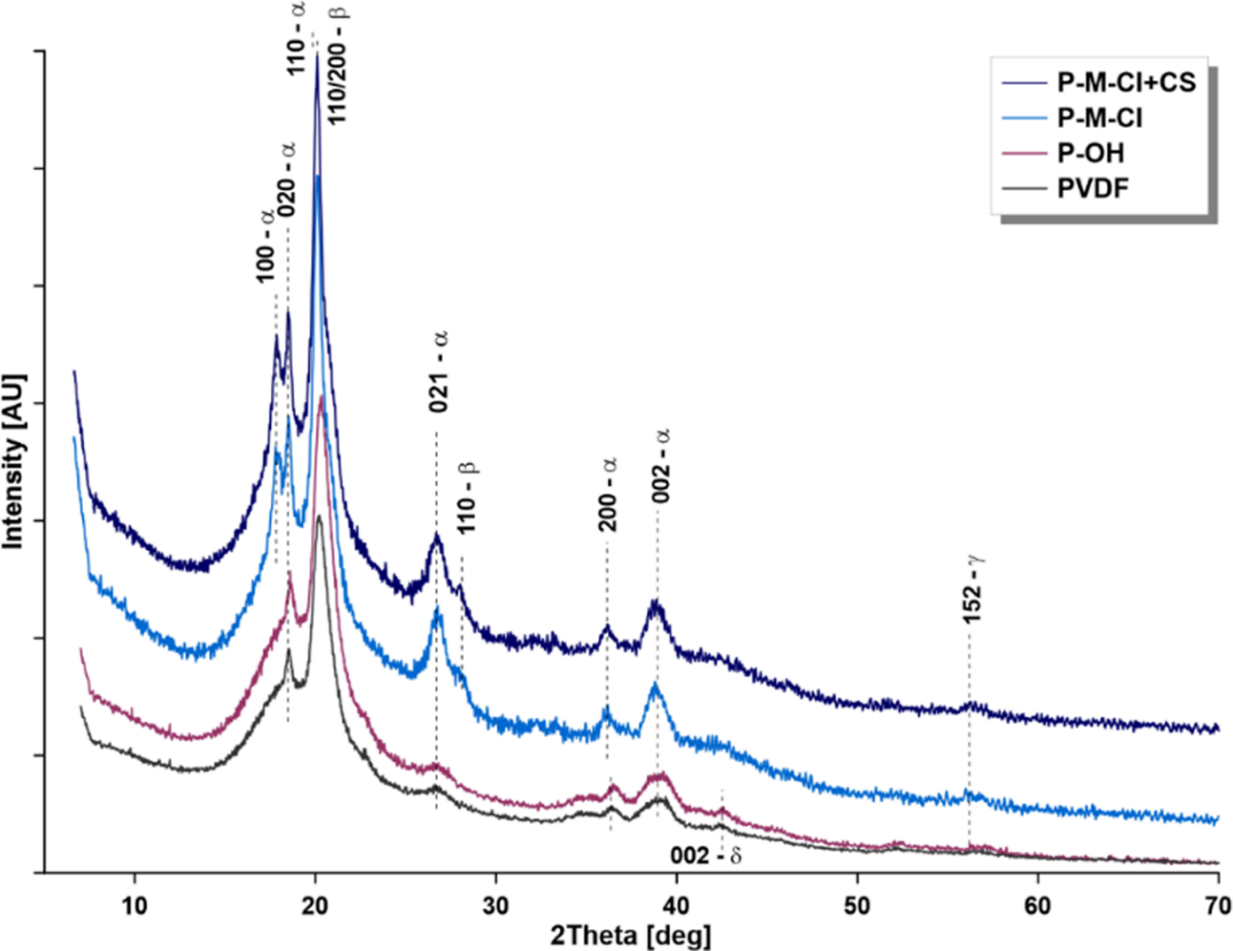
XRD diffractograms of pristine (PVDF), activated (P-OH), and modified samples after silanization (P-M-Cl) and final hybrid material (P-M-Cl + CS).

XRD diffraction was applied to study differences in the phase composition of PVDF-based materials. The main form of PVDF for pristine material was the alpha (α) one. However, after the silanization process and then chitosan attachment, the content of the alpha form changed (Figures 5, S3, Table 1). It was related first to the increase of hydrophobicity owing to the silanization and in the next step to the presence of chitosan. Pristine PVDF membrane was characterized by the ratio of crystallinity degree β/α equal to 0.361 (Table 1). As an effect of the activation process, the reduction of this factor to 0.301was noticed. Furthermore, silanized and hybrid materials possessed a higher crystallinity degree in the range of 0.423–0.437 for silanized materials and 0.426–0.447 for the hybrids. The highest values were found for the materials modified with M-Cl molecules (0.447). Fontananova et al.^46^ presented an example of modification and controlling of phase composition in PVDF by simple salt addition, for example, LiCl, during the dope solution preparation. The finally prepared membrane was characterized by enhanced hydrophilicity related to the increase of β form.^46^ On the XRD spectra, the peak at 2θ equal to 28.2° (Figures 5 and S3) that appeared after modification with a silane-based modifier revealed that chemical interaction took place between the modifiers and the PVDF polymeric backbone.^47^ The appearance of the peak at ca 40° was associated with the occurrence of nanodomains of siloxane, of which the first diffraction peak was at ca. 21.0–21.8°. After the chemical attachment of chitosan, the widening of peaks around 20° was noticed and that was associated with the crystalline structure of chitosan. Pristine chitosan (Figure S3D) possessed a characteristic peak at ca 10°. However, the disappearance of this peak for hybrid material ensured the attachment of the chitosan with high effectiveness.^48^

**Table 1:** Material and Mechanical Characterization of the Investigated Materials

				pore size [μm]					
sample	LEP [kPa]	β/α	*R*_a_ [nm]	min	max	Av.	*F*_a_ [nN]	*E* [GPa]	*E*/*H* [—]
PVDF	146.6	0.361	85	0.09	0.482	0.408	25.20	2.10	16.15
P-OH	227.5	0.301	110	0.31	0.455	0.421	42.30	2.25	10.71
Silanized Samples									
P-M-Cl	164.4	0.437	209	0.23	0.438	0.412	47.32	2.28	10.86
P-M-Cl_DIPEA	187.3	0.423	217	0.21	0.448	0.416	46.32	2.28	10.36
Hybrid Materials									
P-M-Cl + CS	320.9	0.447	260	0.15	0.433	0.367	15.22	2.51	10.04
P-M-Cl_DIPEA + CS	244.9	0.426	228	0.12	0.400	0.333	36.60	2.47	9.88

### 3.4 Hybrid Material Formation—Morphological Study

**Figure 6:**
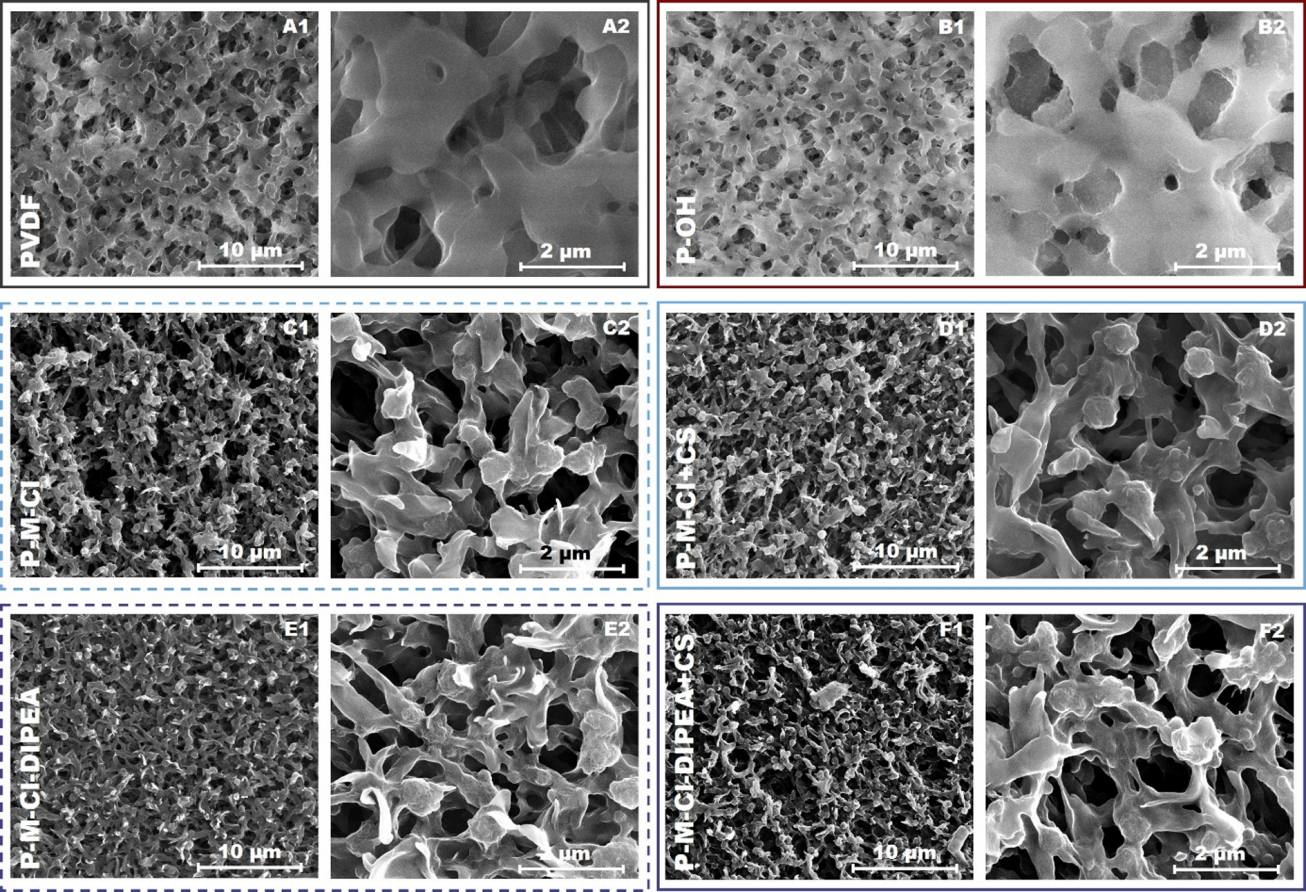
SEM images of the investigated samples. (A1,A2) Pristine PVDF; (B1,B2) activated P-OH; (C1,C2) silanized with P-M-Cl; (D1,D2) hybrid P-M-Cl + CS; (E1,E2) silanized with P-M-Cl-DIPEA; and (F1,F2) hybrid P-M-Cl-DIPEA + CS.

Microscopic study and porosity tests shed light on the morphological changes of the developed materials. After the first step of the membrane treatment, a blister-like structure was observed (Figure 6A1,A2,B1,B2). That observation was in good accordance with the previous results of PVDF treatment with the piranha reagent.^27^ The activation process also influenced the membrane roughness and pore size of the material (Table 1 and Figures S4, S5). An increase of roughness from 85 to 110 nm was noticed. However, the average pore size of the activated materials grew by 4% (Table 1). Membranes modified with silanes possessed meaningfully different features than the activated and pristine ones. The presence or lack of catalysis had a significant impact. Less visible changes were found for materials silanized with M-Cl in the overall volume morphology (Figure 6C1,C2). It was linked to the structure of the modifier and the small amount of the reactive group (1 chlorine group). However, the surface features were more exposed to the modification process, and the roughness increases from 110 nm for the activated sample to 209 and 217 nm for the silanized ones with M-Cl and M-Cl-DIPEA (Figure S6,S7). The reduction of pore size was greater after the treatment of the modifier without catalyst addition (Table 1).

As a result of hybrid material formation, an alteration in membrane morphology was noticed. The general trend exhibits the increase of material roughness in the range of 5–24% depending on the modifier. This variety in the roughness changes is an important outcome for the tuning of the material properties depending on the material application. Furthermore, wing to the introduction of chitosan to the hybrids, the visible flakes on the SEM images (Figure 5D,F) and a slight diminution of the pore size were noticed in the range of 12–25% (Table 1).

### 3.5 Hybrid Material Formation—Mechanical and Nanotribological Properties

#### 3.5.1 Adhesive Properties

**Figure 7:**
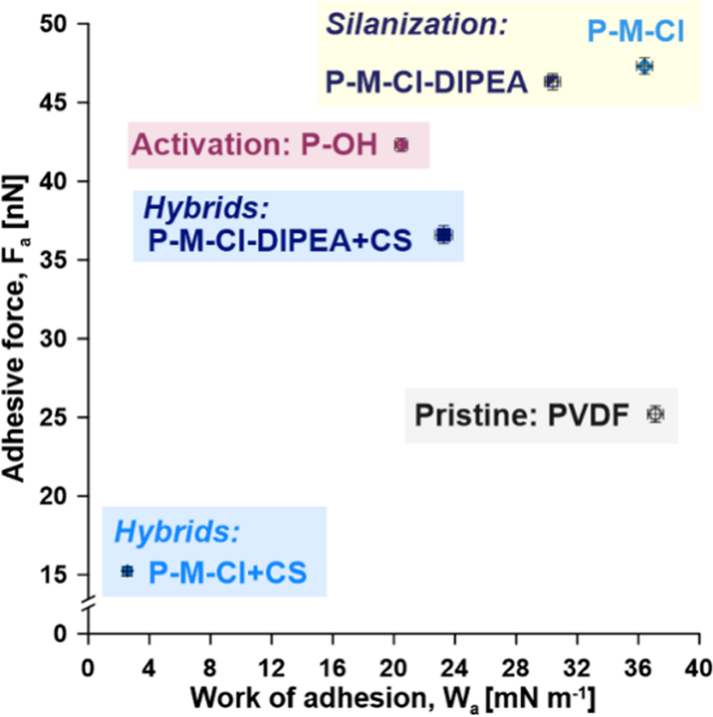
Adhesive properties: adhesive force (*F*_a_) in the function of the work of adhesion (*W*_a_).

The stability of the prepared materials was assessed by the following parameters: nanohardness (*H*), plasticity index (*E*/*H*), Young modulus (*E*), and the correlation between work of adhesion (*W*_a_) and adhesive force (*F*_a_) (Figure 7, Table 1). At the equilibrium of *W*_a_, mechanical properties and wettability possess a critical influence from a thermodynamic point of view.^49^ This is related to the formation and elimination of interfacial areas causing alteration of reversible free energy. Heterogeneities of the surface material, particularly the hybrid one, in nano and/or micro-scale, can let water penetrate across these structures. Finally, nano- or microdroplets can be formed, even on the highly hydrophilic/hydrophobic material.^50^ Such behavior has been found on the prepared membranes, silanized as well as hybrid materials, exhibiting roughness parameters in the range of 85–260 nm (Table 1). In the case of hybrids of hydrophobic materials with hydrophilic domains, the local adhesive features are very important and informative, and these results are gathered in Figure 7. The highest impact of the modification was observed for the hybrid sample of P-M-Cl + CS, *W*_a_ which dropped from 37.12 mN m^–1^ for PVDF to 2.55 mN m^–1^ for the mentioned hybrid. On the other hand, for the same sample, the *F*_a_ changed from 25.2 to 15.22 nN after the introduction of chitosan. It was interesting to observe an increase of local adhesive properties after the first step of modification, that is, silanization. Moreover, even when the properties are studied on a nanoscale (by nanoindentation), the impact of the type of modifier was clearly visible. Higher effectiveness, expressed by a reduced adhesive force and work, was noticed for the surface modified with NCO-M-Cl molecules. It was noticed that by the introduction of the catalyst, it was possible to tune the final adhesion of the materials (Figure 7). The highest values for both, *W*_a_ and *F*_a_, were observed for the P-M-Cl and P-M-Cl-DIPEA. The adhesion work for hybrids was much smaller than for samples after silanization, owing to the higher hydrophobicity level (Figure 7 and Table 1). Such relation was presented by Bhushan and Liu^51^ for the self-assembly monolayers generated from biphenyl thiol and alkylthiol having different reactive groups. The authors showed that the film made from hexadecane thiol with a methyl terminal group was characterized by the lowest frictional force and the *F*_a_ owing to its low *W*_a_ and its very acquiescent long carbon chain. The correlation between *W*_a_ and *F*_a_ on the surface of coated glass and silicon has been studied by Psarski et al.^52^ The most valuable results from the application point of view were to establish the material with a minimal value of work of adhesion as well as adhesion force. This particular outcome has been achieved by attaching chitosan particles to the already silanized membranes. The values of the adhesive force diminished from 47.32 to 15.22 nN, and the work of adhesion changed from 36.4 to 2.55 mN m^–1^, respectively (Figure 7).

#### 3.5.2 Mechanical Resistance—Nanotribology

An essential matter in the evaluation of novel materials is the assessment of their mechanical properties. In the case of molecular decoration, it is much more important to analyze the mechanical resistance in nanoscale. To achieve this aim, the nanotribological study was performed by the utilization of the nanoindentation method. The values of Young modulus (*E*), adhesion force (*F*_a_), and plasticity index (ratio of Young modulus and nano-hardness—E/H) were calculated and gathered in Table 1. From the obtained data, it was possible to evaluate if the membranes will be stable during the further application in the separation process considering the risk of membrane damage owing to the potential stress of the material in compression during the process. As already presented, the formation of silanized surfaces and then materials possessing hydrophilic islands on the hydrophobic matrix reduced significantly the adhesion (Table 1). Not only the changes in the adhesive features but also the changes in the mechanical ones have been detected. The changes in the nanoscale caused by implementing nanotribological measurements revealed that much more resistant materials with a higher level of Young modulus and lower plasticity index have been developed, particularly after the chemical bonding of chitosan molecules. The elasticity factor was enhanced by 50% as an effect of activation. In the case of silanization and hybrid formation, the improvement in the elasticity was 49–56 and 61–64%, respectively. The comparison has been done in the reference to pristine PVDF (Table 1). Better improvement was noticed for the samples with the presence of a catalyst. The described trend was in good accordance with the work of others. Zeng and co-workers^53^ generated PVDF nanocomposites with the addition of fluoropropyl polyhedral oligomeric silsesquioxane (FP-POSS). The additives varied from 0 to 8 wt %. The authors found that the best upgrading of the mechanical features equal to 6% was for sample with 3 wt % of FP-POSS in PVDF. The differences in the elasticity index in our work was in the range of 7–9.5% for activated and hybrid membranes, respectively. The slightly lower values, that is, 7% after activation and 8% after functionalization, were observed in our previous work,^15^ where the functionalized chitosan were used to the membrane modification. The gathered data in the current work ensure that it is possible to generate stable and resistant membranes *via* hybrid formation.

### 3.6 Hybrid Material Formation—Wettability Study

The material wettability is an essential factor in the evaluation of material utility as well as resistance to fouling and damaging the surface. In defining the wettability, the level of hydrophobicity or hydrophilicity must be measured. Membrane wetting can take place by the accumulation and adsorption of an amphiphilic particle, lipids, proteins, or fat on the membrane surface.^18^ The attraction between these molecules and the membrane decreases the surface tension of liquid at the membrane surface, which in turn diminishes the wetting pressure directly related to the critical surface tension.^54−56^ If the wetting pressure is smaller than the gradient of operating pressure, the liquid can enter the membrane pores. Membrane wetting upsurges the resistance of mass transfer of membrane because the diffusivity in the liquid phase is smaller than the vapor phase. The critical situation is that when the membrane is completely wetted out, water molecules would not diffuse in vapor form anymore.

Moreover, it should be pointed out that material features, for example, chemistry, phase composition, and roughness, possessed a significant influence on the final hydrophilicity/hydrophobicity of the material, principally on PVDF-based samples.^22,57,58^ Meringolo et al.^59^ presented the sustainable method of PVDF membrane preparation by merging vapor-induced and liquid-induced phase separation techniques in a controlled way, which made possible the preparation of symmetric porous membranes with customized rough surface topography and hydrophobicity. For instance, just by changing the humidity during the membrane formation process from 50 to 64%, it was possible to turn the hydrophobic (CA = 140° and roughness = 670 nm) materials to the hydrophilic (CA = 75° and roughness = 340 nm) ones. The same tendency of increasing hydrophobicity with the higher roughness parameters was presented, for instance, by Rezaei et al.^60^ It should be remembered that if two surfaces having different hydrophobicity are roughened, both can be turned to a superhydrophobic state.^61^

**Figure 8:**
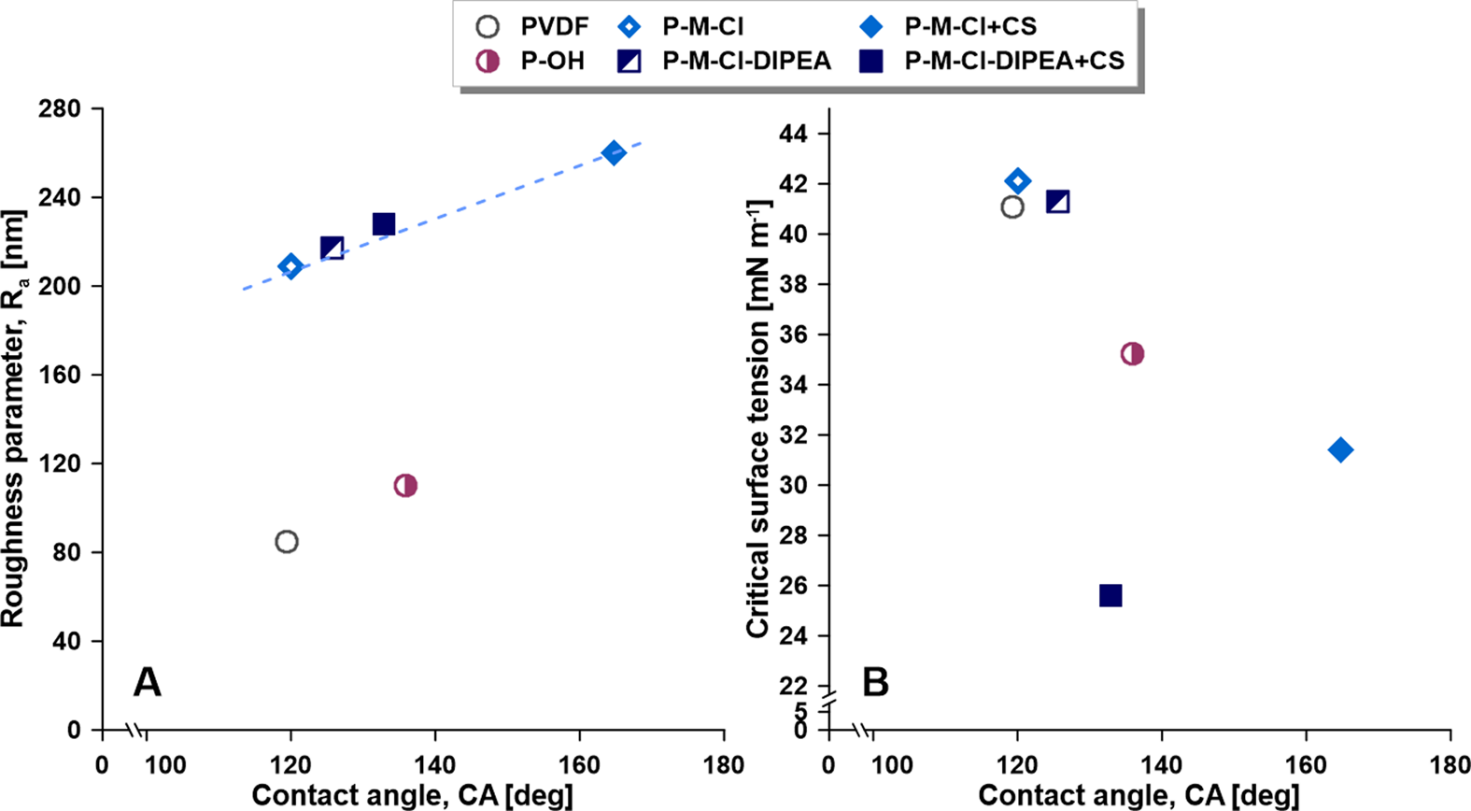
Relation between CA and roughness parameters (A) and critical surface tension (B).

In our work, a very effective method inspired by nature has been developed. The two-step procedure made it possible to tune both CA and roughness in an easy way. First, the activation process with piranha reagent produces the highly reactive, hydroxyl-rich surface with high CA (PVDF: 119.4 ± 1.1° and P-OH: 135.9° ± 1.2°) and improved roughness angle (PVDF: 110 nm and P-OH: 85 nm) (Figure 8). In this particular case, the high level of hydrophobicity was associated with the roughness (Figures S4 and S5) from one side and from the other one with the chemistry, that is, phase composition of the sample (Figure 5). The first step of modification with silane-based modifiers gave the materials with higher roughness, particularly with the treatment with the presence of a catalyst (Figure 6E). However, the level of hydrophobicity was smaller than for the activated one (Figure 8). The most interesting part of the obtained wettability features was observed for the hybrid materials after the chemical attachment of the chitosan particles (Figure 6). During the hybrid formation without the presence of DIPEA, a material with a CA equal to 164.8° ± 0.8° and a roughness of 260 nm was generated. However, when the synthesis was accomplished with the addition of the catalyst, roughness and CA were slightly improved (Figure 8). The data are in accordance with the Cassie–Baxter’s wetting model,^62,63^ foreseeing that for a heterogeneous hydrophobic surface, a nonwetting liquid may not enter into surface cavities, resulting in the generation of air pockets, leading to a composite solid–liquid–air interface where surface roughness rises with the hydrophobicity. The mentioned behavior was proven by the linear relation between these two factors for all modified samples.

The resistance of wettability was also assessed by the critical surface tension, which defines the limit of surface wettability. All of the liquids possessing a value of surface tension lower than the critical surface tension of the material will wet the surface. This is crucial from the application point of view in order to produce the material with the possible low value of γ_cr_ to cover the broad range of the liquids that might have contact with the membrane without the risk of the wetting just limiting the separation across the material. In Figure 8B, the correlation between the level of hydrophobicity and γ_cr_ is presented. The slightly higher value of the critical surface tension of the silanized materials was related to the presence of −NCO groups and different chemistry, in comparison to pristine and activated PVDF materials. However, the substantial reduction of critical surface tension values for the hybrid materials (P-M-Cl-DIPEA + CS and P-M-Cl + CS) was owing to the chitosan molecules and their character. Even though chitosan with hydrophilic characteristic was attached, the value of γ_cr_ was diminished (Figure 8). This outcome verified that chitosan-like island structures were produced. Additionally, it is shown that more influential for SFE and γ_cr_ is surface chemistry than its morphology.^15^

The overall SFE (Figure S8) changed in the following way: 29.52 ± 0.88, 23.35 ± 0.70 mN m^–1^, for the PVDF and P-OH. Then, a slight improvement was observed as an effect of the silanization process, 28.43 ± 0.50 mN m^–1^ (P-M-Cl) and 30.70 ± 0.65 mN m^–1^ (P-M-Cl-DIPEA). Finally, the hybrids possessed the value of total SFE equal to 20.51 ± 0.65 mN m^–1^ (P-M-Cl + CS) and 34.34 ± 0.48 mN m^–1^ (P-M-Cl-DIPEA + CS). The polar part of the SFE was in the range of 9 and 25% of total SFE depending on the sample (Figure S8). However, the small impact of polar interaction ensures these materials’ suitability to the membrane distillation process.

The differences in the wettability of the material were also observed in the LEP values (Table 1). For the non-modified material, that is, PVDF, the value of LEP was equal to 146.6 kPa that is comparable to the literature data. AlMarzooqi and co-workers^26^ have shown that LEP for PVDF membranes varied between 96 and 167 kPa and was dependent on the membrane formation conditions (humidity, time, temperature, and the concentration of PVDF). Nevertheless, the activated and hybrid materials were characterized by LEP higher than the pristine sample. P-OH was characterized by 227.5 kPa, but the silanized membranes possessed LEP on the following level 164.4 kPa and 187.3 kPa for P-M-Cl and P-M-Cl-DIPEA, accordingly. The upsurge of the LEP for modified and hybrid materials referred to neat PVDF needs to be connected to smaller value of the maximum pore size. This phenomenon favors higher LEP.^15,26,60^ The obtained results confirmed that the developed method allows to generate more water-resistant membranes (Table 1).

### 3.7 Water Transport across the Membranes

**Figure 9:**
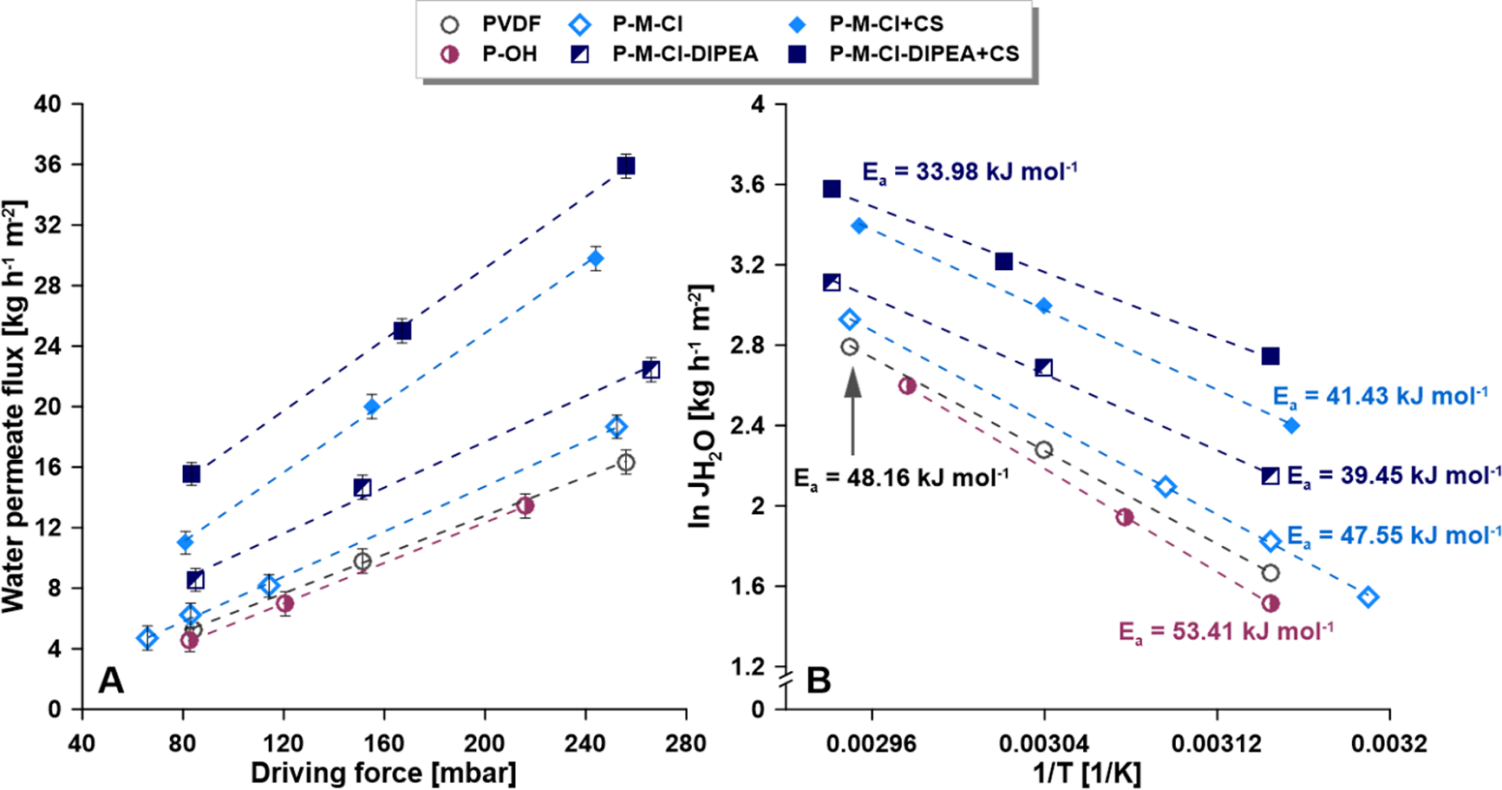
(A) Permeate flux of water in the function of driving force and (B) *E*_a_ for water transport across the membranes.

Based on the collected results from the systematic material study (Figures 3–8), it was verified that fabricated separation membranes are appropriate for MD process. The generated membranes fulfilled all the requirements of materials for MD, that is, the materials are porous (Table 1), hydrophobic (Figure 8A), and no wetting behavior (Figure S9) was revealed. In the first step of membrane application, transport features, that is, water transport across the membrane, were determined. To do so, three different driving forces were used to evaluate the (*E*_a_) (eq 3).^15,28^ It should be stressed that activation energy is a complex factor.^28,30,31,64^ Taking into account the *E*_a_, a surge of the activation energy for transport of water from 48.16 kJ mol^–1^ (PVDF) to 53.41 kJ mol^–1^ (P-OH) was observed after material activation. It can be explained by the production of a stronger hydrophobic barrier (Figure 8), creating the water transport limited. Subsequently, owing to the silanization process as well as hybrids containing chitosan materials, a substantial reduction of activation energy was noticed (Figure 9). It was particularly interesting to observe the influence of the presence of catalysts on that physicochemical factor, that is, *E*_a_. For the materials synthesized with the addition of the catalyst (DIPEA), ca. 22% improvement was observed in the water transport. The values of the activation energy were smaller for P-M-Cl-DIPEA (39.45 kJ mol^–1^) and P-M-Cl-DIPEA + CS (33.98 kJ mol^–1^) in comparison to P-M-Cl (47.55 kJ mol^–1^) and P-M-Cl + CS (41.43 kJ mol^–1^), respectively. Furthermore, the observed lower values of *E*_a_ for the hybrid materials was associated with the chitosan attachment and production of stable hydrophilic layer on the membrane. The determined values of *E*_a_ for all membranes were in good agreement with the data from other PVDF-modified membranes.^15,27^

**Figure 10:**
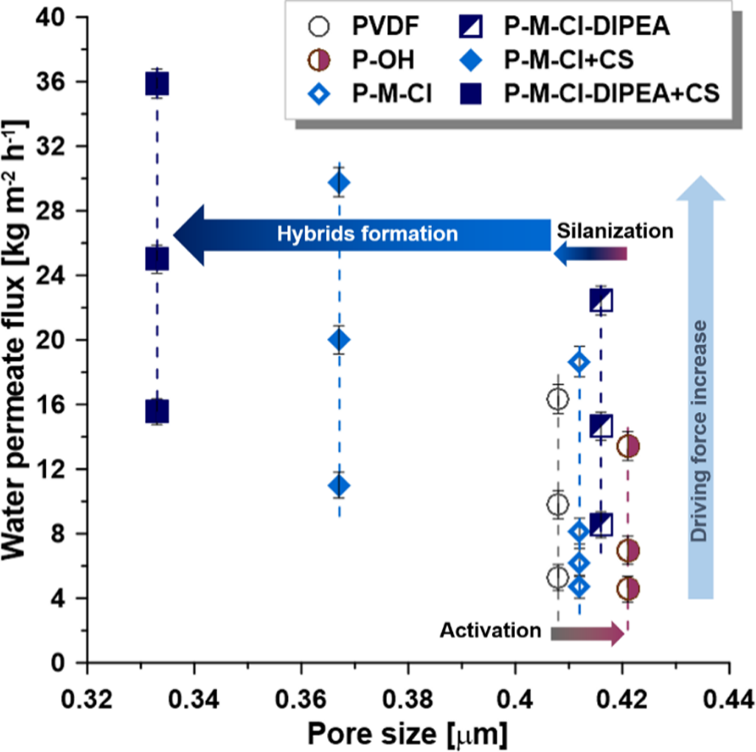
Relation between transport properties and pore size after activation, silanization, and hybrid formation.

The hydroxylated membrane (P-OH), owing to its characteristic (Figure 8), was less permeable than a pristine one (Figure 8). It was related not only to the hydrophobicity level but also to the roughness and pore size of the materials (Figure 10). The improvement in the transport properties for the silanized materials in comparison with the activated ones was in the range of 16–39 and 39–61% for the P-M-Cl and P-M-Cl-DIPEA, respectively, depending on the driving force. However, substantial differences have been noticed after the introduction of chitosan particles. In this situation, the enhancement was 221–286% for the P-M-Cl + CS and 261–356% for P-M-Cl-DIPEA, accordingly. Such improvement was possible to obtain only owing to the chitosan presence and their chemistry, making the final material much more permeable for vapors of solvent, that is, water. For all investigated samples, a linear correlation between driving force and water flux was observed (Figure 9A,B). Additionally, all the created membranes have been characterized by outstanding stability during the MD process. The lack of flux reduction verified that no wetting occurred in the course of AGMD (Figure S9).

For the full and systematic characterization of the water transport across the prepared membranes, the overall transport coefficient was determined (eqs 4 and 5). The data are in good accordance with other results presenting the consistent membrane characterization, gathered in Table S1. The highest values of the transport coefficient, *K* parameter (eq 5), were found for the hybrid materials, particularly for P-M-Cl-DIPEA + CS. The physicochemical parameters, for example, hydrophobicity level, roughness, pore size, γ_cr_, and SEP, have a critical influence on the overall transport features. In the presented research, the measured values assessing the transport were improved in the comparison to other works.^27^ For the PVDF membrane modified with fluorinated and non-fluorinated silane modifiers, the permeance coefficient, depending on the grafting agent, ranged between 38.4 and 40.5 kg h^–1^ m^–2^ bar^–1^.^27^ In our current work, the permeance coefficient was placed between 90.2 and 304.4 kg h^–1^ m^–2^ bar^–1^.

### 3.8 Desalination Process—Air Gap Membrane Distillation

**Figure 11:**
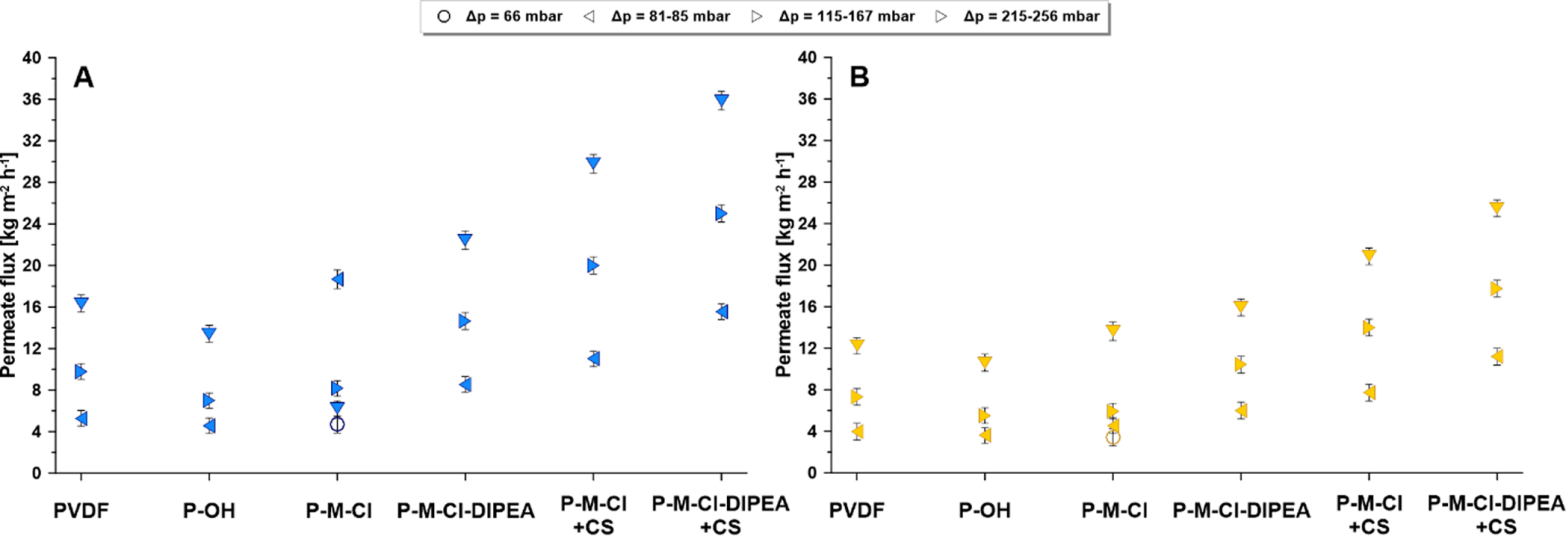
Transport properties—(A) feed: water and (B) feed: 0.5 M NaCl across the investigated membranes under different driving forces.

**Figure 12:**
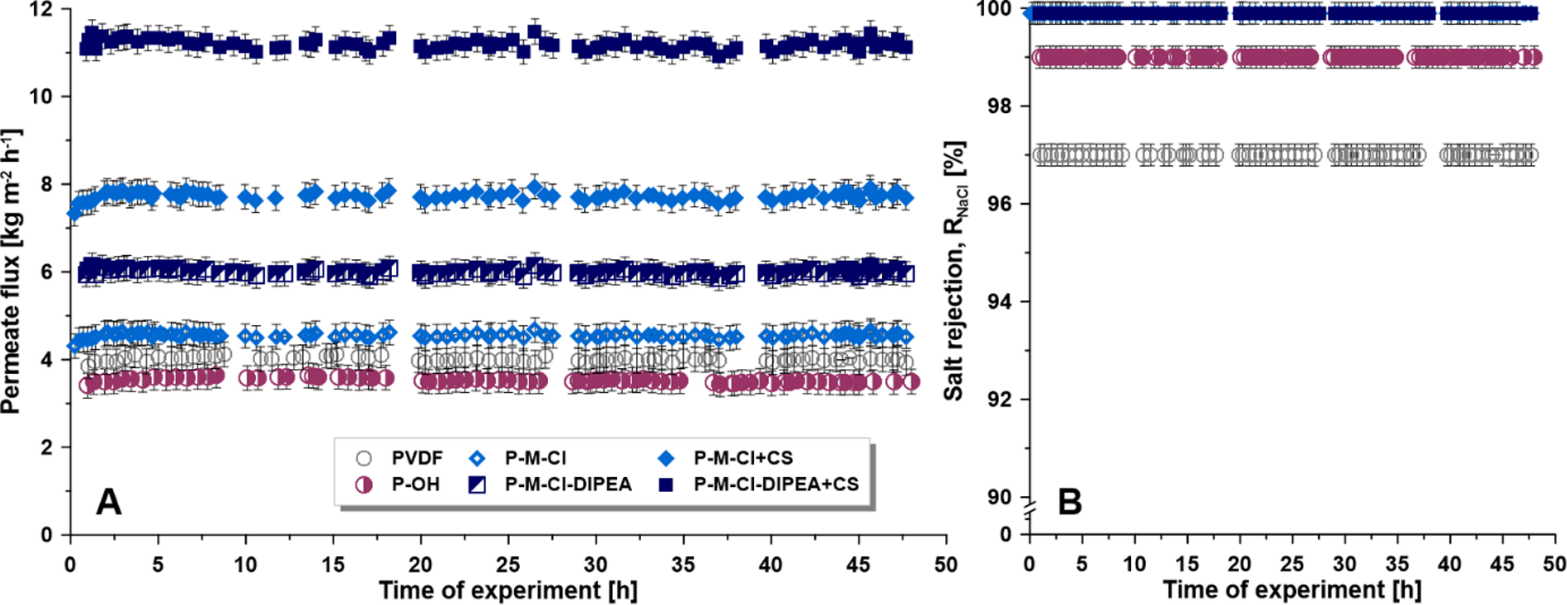
Membrane stability: the evolution of permeate flux (A) and salt rejection (B) during the long-term desalination process (driving force 81–85 mbar).

After the detailed characterization of transport of water across the generated materials, the desalination process was performed. In Figure 10, the comparison of permeate fluxes when pure water (Figure 10A) and salty water (Figure 11B) were used as a feed solution for various driving forces has been presented. The concentration of salty water was selected similar to seawater.^65^ The observed lower value of the permeate flux for the salty water as a feed is related to the fact that only vapors of solvents can be transported through the hydrophobic pores of the membrane in the membrane distillation process. In the previous sections of the work, it was presented that these conditions are fulfilled. Specifically, all membranes were porous and hydrophobic. The diminution of transport features for the NaCl solution is controlled by Raoult’s law.^66,67^ The transport of solvent vapors in MD is relative to the difference of water vapor pressure between the feed and the permeate calculated basing the eq 2. For that reason, the increase of transport properties with the increase of driving force was observed, independent of the used feed (Figure 11). Generally, the lower value of the permeate flux was noticed for the activated membrane that was related to its chemistry. Furthermore, an interesting observation was found for the modified membranes. A gradual increase of the transport features was noticed after the first step of modification, that is, silanization process. Subsequently, more significant improvement has been observed after hybrid formation, a hydrophobic surface possessing hydrophilic islands. The permeate flux was enhanced from 3.60 ± 0.11 kg m^–2^ h^–1^ for P-OH membrane to 11.18 ± 0.17 kg m^–2^ h^–1^ for P-M-Cl-DIPEA + CS when 0.5 M NaCl was used as a feed (Figure 11). To evaluate the membrane stability, all membranes were tested in runs of ca. 50 h (Figure 12A), with pure water and 0.5 M NaCl aqueous solutions. The data for the selected driving force is presented in Figure 11. Moreover, the salt rejection coefficient (*R*_NaCl_) was monitored (Figure 12B). For all membranes, the values of R_NaCl_ were close to unity, ensuring no leakage in the course of AGMD. Furthermore, the stability of the membranes was proven by unchanged values of the flux in long-time measurements (Figure 12). The slightly reduced value of *R*_NaCl_ for the activated membrane (P-OH) might be associated with small alterations in material properties (CA, root mean square, and critical surface tension).

The obtained data demonstrated the high effectiveness of the generated materials possessing very good permeability and salt rejection. Based on the literature survey, it can be concluded that the developed method of hybrid membrane formation having a hydrophobic matrix with hydrophilic islands makes it possible to prepare promising separation materials. Xu and co-workers^68^ presented an example of polyamide nanofilm composite membranes supported by chitosan-coated PVDF nanofibrous 16.5 L m^–2^ h^–2^ and salt rejection of 94.4%; however, chitosan was only added as one of the layers, without any chemical changes. Al-Mubaddel et al.^69^ have shown the separation material also using the nanofiber structure membrane. The work described the production of nanofiber membranes from PVDF coated with chitosan to enhance membrane properties such as hydrophilicity, mechanical properties, water flux, and salt rejection. The authors showed that by adjusting the type of solvents, that is, tetrahydrofuran (THF) and *N*,*N*-dimethylformamide (DMF), it was possible to improve transport features. Membranes produced without THF were very stable and more permeable with the highest value of the water flux equal to 52.4 L m^–2^ h^–2^. However, the salt rejection during the experiment with this membrane was 6.65%. After the application of chitosan coating by immersion method, the flux reduced to 28.5 L m^–2^ h^–2^ and rejection rose to 28.6%. The observed low-membrane performance was related mostly to the low value of the CA equal to 70°.^69^ None of the published research focused yet on the PVDF membrane preparation with the chemically attached chitosan.

The hybrid materials with chemically anchored chitosan might have important implications for other water treatment processes, for example, for the removal of volatile organic compounds or filtration process. Moreover, owing to the dual nature, that is, combining hydrophobic and hydrophilic feature, the material can be applied for the separation of water–oil mixtures.

### 3.9 Stability

**Figure 13:**
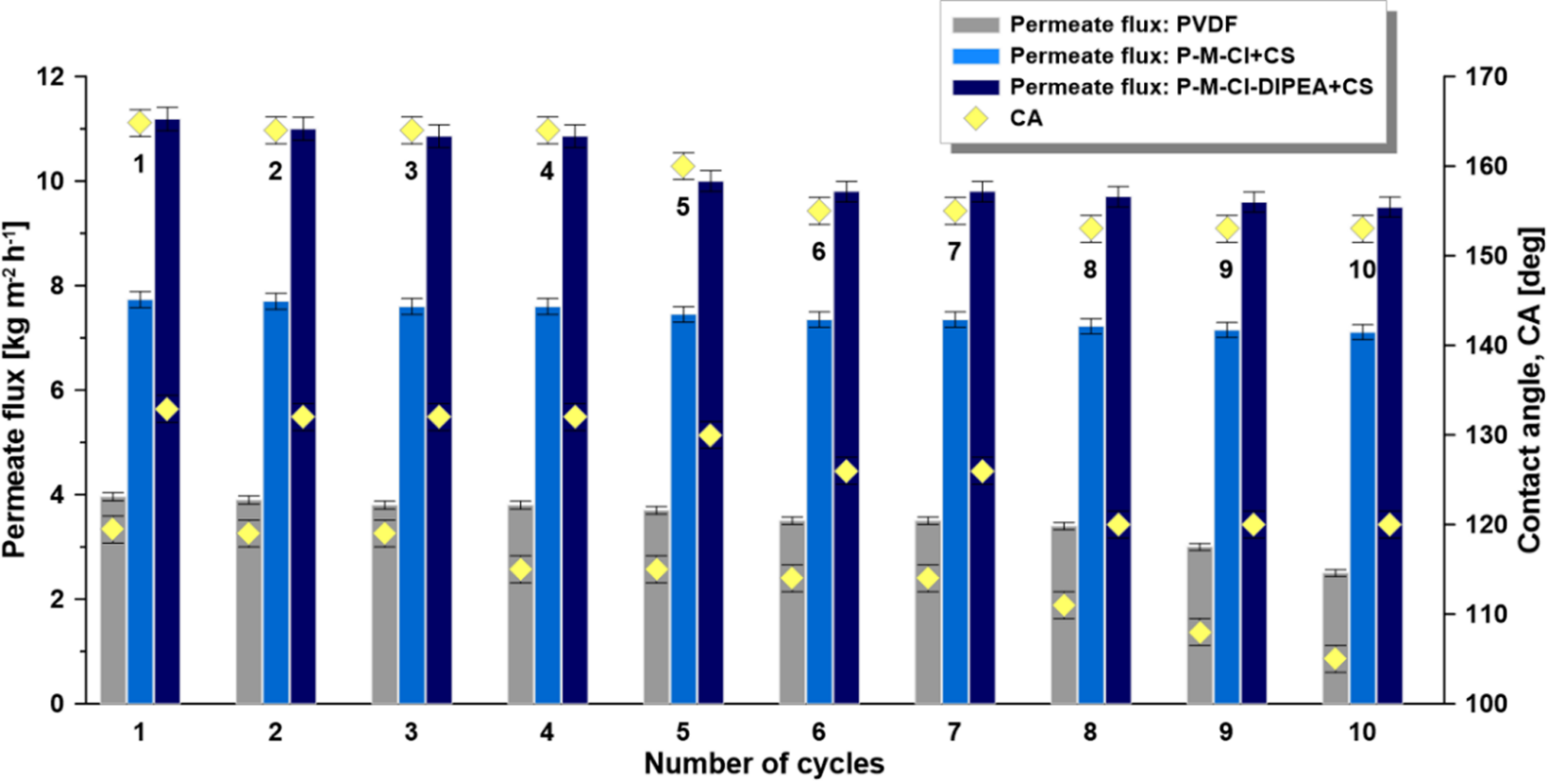
Membrane stability during the desalination processes.

In the final step, the membrane stability and utility in the desalination process was determined during 10 cycles of membrane distillation processes. Each run lasted for ca. 40–45 h. During the experiments, permeate flux, salt rejection coefficient, and value of CA for pristine PVDF and both hybrids, P-M-Cl + CS and P-M-Cl-DIPEA + CS, were monitored (Figure 13). All membranes were characterized by a very good stability. However, the diminution of flux and CAs of about 35 and 14%, respectively, were observed for the PVDF membrane. The hybrid materials containing chitosan were much more stable with the reduction of permeate flux on the level of 8% for P-M-Cl + CS and 15% for the P-M-Cl-DIPEA + CS sample, accordingly. The CA change for the hybrid materials was about 7 and 10%. Furthermore, there was no influence of the long-term utilization of the membrane on the salt rejection coefficient.

## 4 Conclusions

The efficient method of modifying fluoropolymer membranes by the formation of hybrid hydrophobic/hydrophilic materials was presented. In the developed method, the engineering of the desert beetle’s armor possessing a hydrophobic surface with hydrophilic islands was biomimicked. To form hydrophilic domains, biowaste, a natural polymer, chitosan was used and covalently attached *via* silane linkers to the membrane. The presence of chitosan significantly improved membrane stability and effectiveness in the course of the desalination process. The substantial enhancement of water transport across the membranes was related to the introduction of hydrophilic chitosan, promoting transport. The high efficacy of the process was verified by the set of advanced techniques giving the holistic image of functionalized materials. FTIR–ATR, XRD, and SEM confirmed that the functionalization of chitosan took place *via* its amine and hydroxyl groups and terminal isocyanate groups from the modifier attached to the PVDF membrane. Application of the linker’s molecules with highly reactive chlorine moieties allowed made it possible to generate a highly organized surface. The element of novelty was to present the modification route with a catalyst. Thanks to the introduction of the catalyst, it was possible to tune the final adhesive of the materials as well as their hydrophobicity and roughness features. The hydrophobicity level of the hybrid materials rose from 119.4° for pristine PVDF to 164.8° for P-M-Cl + CS and 132.9° P-M-Cl-DIPEA + CS, respectively. However, the work of adhesion was reduced to 2.55 mN m^–1^ for P-M-Cl + CS and 23.26 mN m^–1^ for P-M-Cl-DIPEA + CS. The value of the work of adhesion for the pristine membrane was equal to 37.10 mN m^–1^. Moreover, the membranes modified in the presence of catalysts were more permeable. Water flux increased from 5.28 to 11.00 and 15.55 kg m^–2^ h^–1^ for membrane with chitosan and membrane with chitosan modified under the presence of the catalyst, respectively.

## Supporting Information Available

ATR–FTIR spectra of the pristine material and the activated one; modifier (M-Cl) and catalyst (DIPEA) molecules; XRD patterns; pristine (PVDF), activated (P-OH) and modified samples, after silanization (P-M-Cl-DIPEA), and the final hybrid material (P-M-Cl-DIPEA + CS); pattern of chitosan; AFM images of the pristine PVDF membrane, high phase and amplitude images in the 2D and 3D projection; AFM images of the activated PVDF membrane (P-OH), high phase and amplitude images in the 2D and 3D projection; AFM images of the silanized PVDF membrane (P-M-Cl), high phase and amplitude images in the 2D and 3D projection; AFM images of the hybrid PVDF membrane (P-M-Cl + CS), high phase and amplitude images in the 2D and 3D projection; SFE with dispersion and polar components; water flux stability during the AGMD process, pristine membrane PVDF, activated PVDF (P-OH), silanized membrane (P-M-Cl), silanized membrane in the presence of catalyst (P-M-Cl-DIPEA), hybrid PVDF membrane (P-M-Cl + CS), and hybrid PVDF membrane prepared in the presence of a catalyst (P-M-Cl-DIPEA + CS); and water transport features of the investigated membranes (PDF)
