# Supplementary material for: How Can the Desert Beetle and Biowaste Inspire Hybrid Separation Materials for Water Desalination?
Source: ACS Appl Mater Interfaces. 2021 Mar 1;13(9):11268–83. doi: 10.1021/acsami.0c21649 (PMC8031369; doi:10.1021/acsami.0c21649)
Supplement: Supplementary file 1 — am0c21649_si_001.pdf [file am0c21649_si_001.pdf]

Supporting information

How Can the Desert Beetle and Biowaste Inspire Hybrid Separation Materials for Water Desalination?

Samer Al-Gharabli[1)], Bana Al-Omari[1)], Wojciech Kujawski[2)], Joanna Kujawa[2)*]*

[1)] Pharmaceutical and Chemical Engineering Department, German Jordanian University, Amman 11180, Jordan

[2)] Faculty of Chemistry, Nicolaus Copernicus University in Toruń, 7 Gagarina Street, 87-100 Toruń, Poland

Corresponding authors:

Emails:

Samer Al-Gharabli: samer.gharabli@gju.edu.jo

Joanna Kujawa: joanna.kujawa@umk.pl

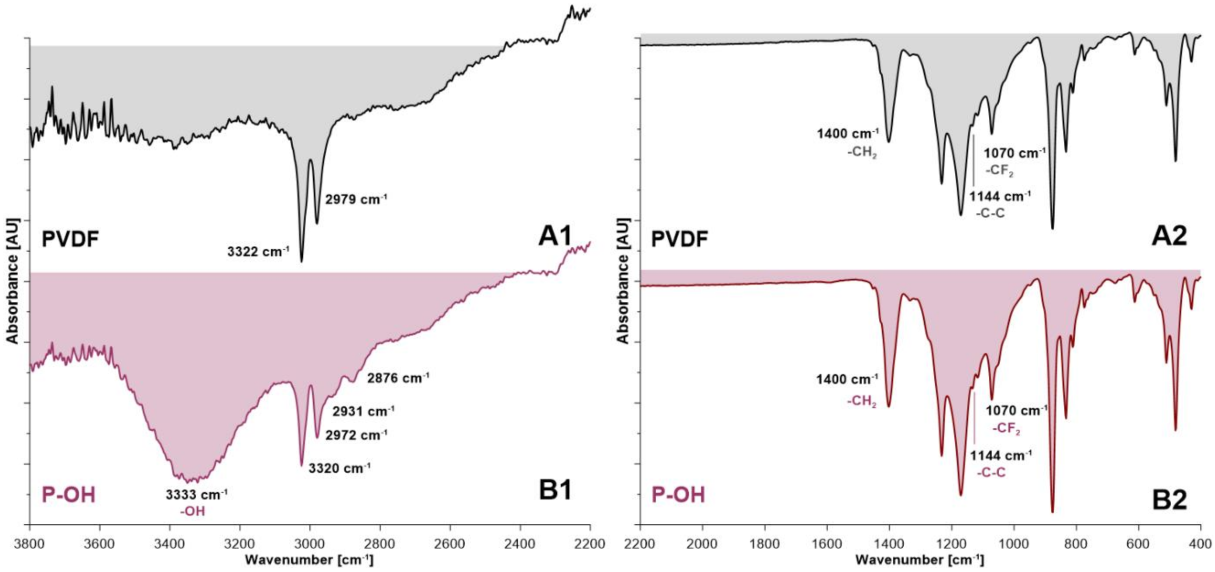

Figure S1. ATR-FTIR spectra of pristine material (A1, A2) and activated one (B1, B2)

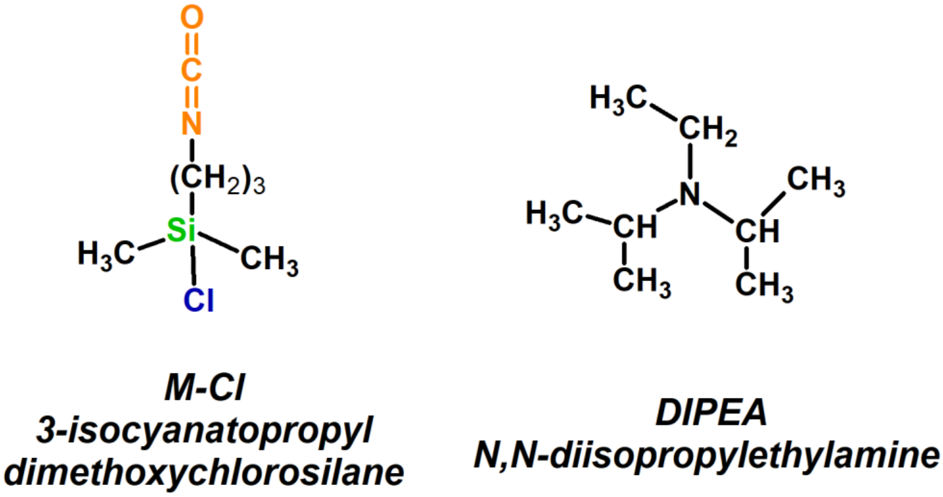

Figure S2. Modifiers (M-Cl) and catalysts (DIPEA) molecules.

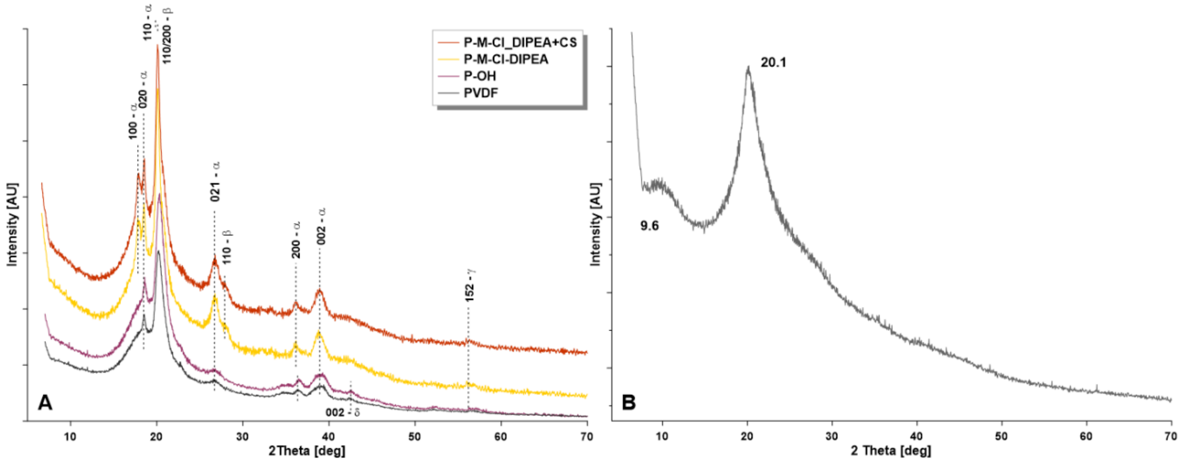

Figure S3. XRD patterns. Pristine (PVDF), activated (P-OH) and modified samples, after silanization (P-M-Cl-DIPEA) and final hybrid material (P-M-Cl-DIPEA+CS) (A). Pattern of chitosan (B).

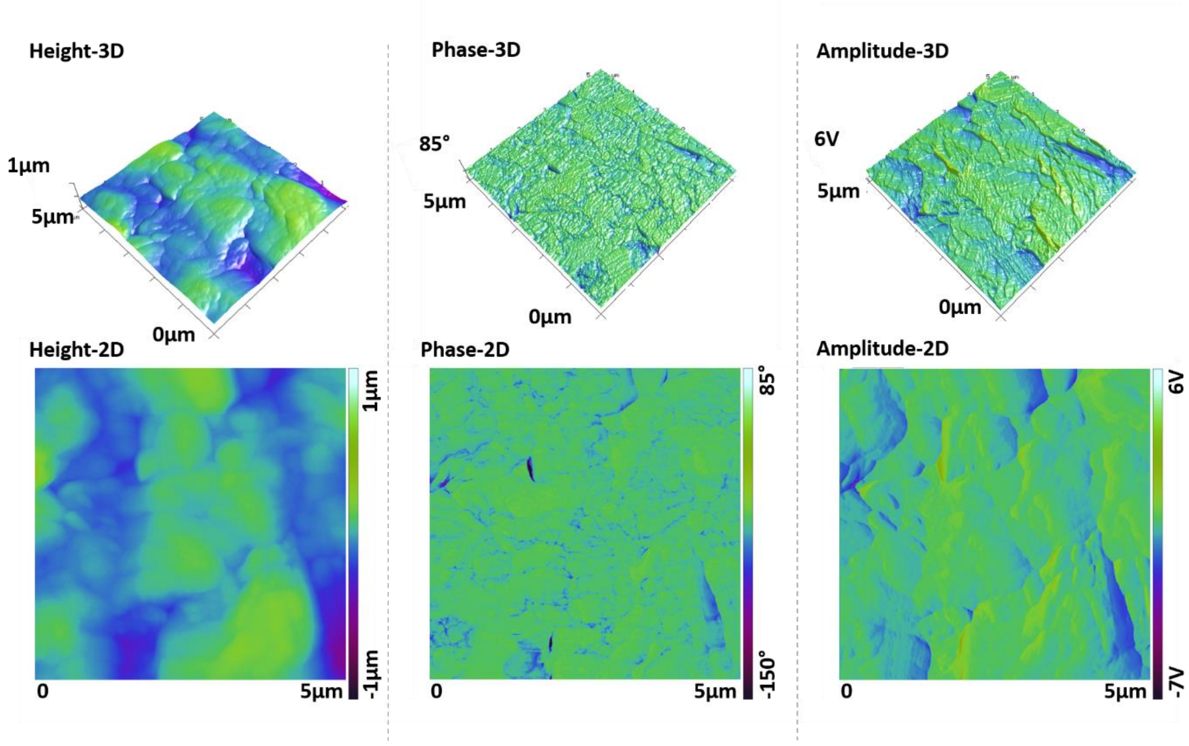

Figure S4. AFM images of pristine PVDF membrane, high, phase and amplitude images in the 2D and 3D projection.

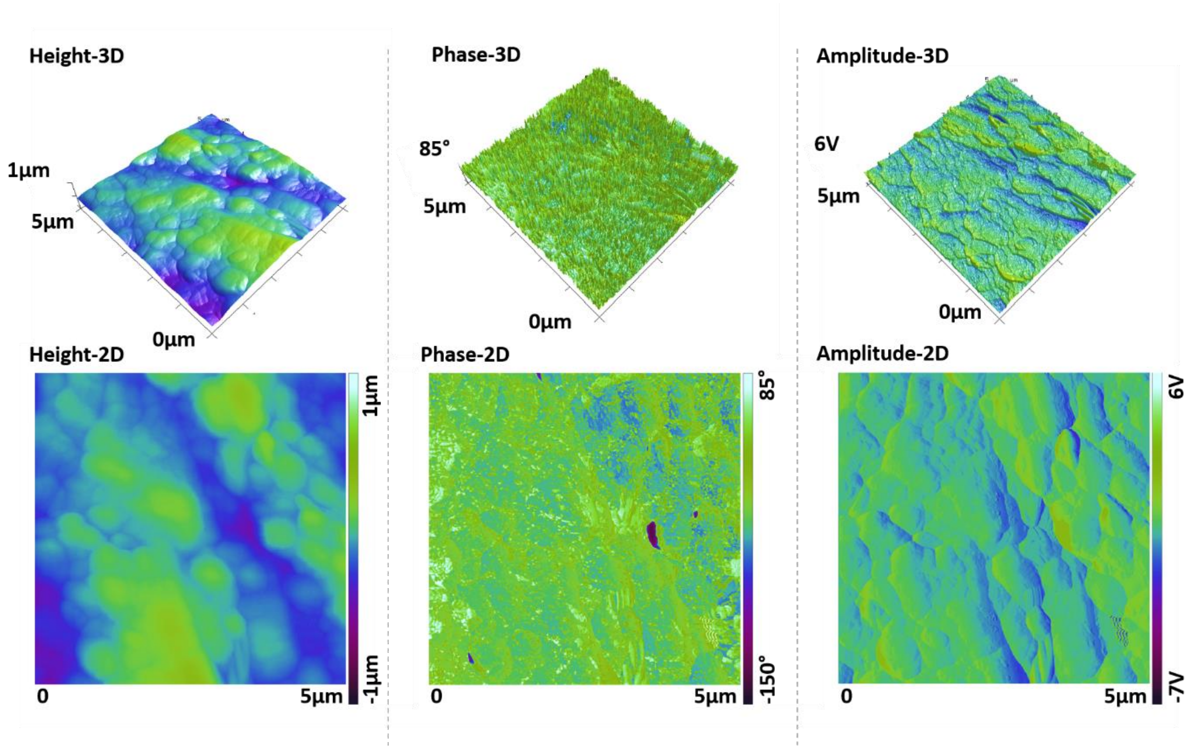

Figure S5. AFM images of activated PVDF membrane (P-OH), high, phase and amplitude images in the 2D and 3D projection.

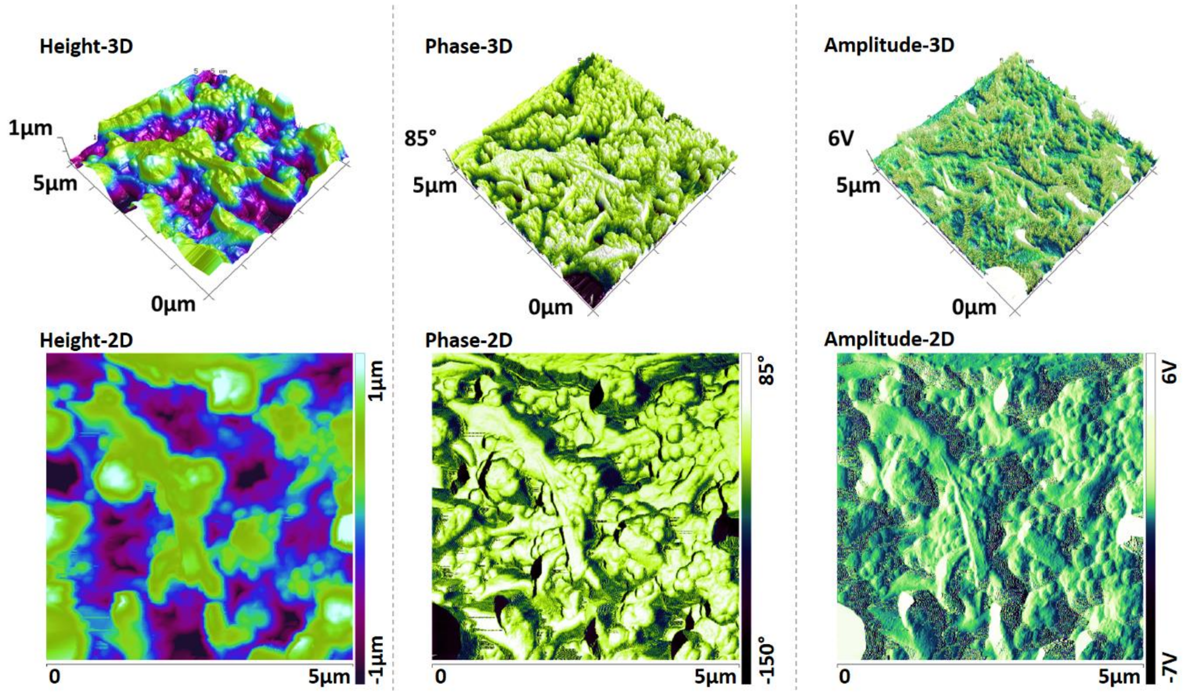

Figure S6. AFM images of silanized PVDF membrane (P-M-Cl), high, phase and amplitude images in the 2D and 3D projection.

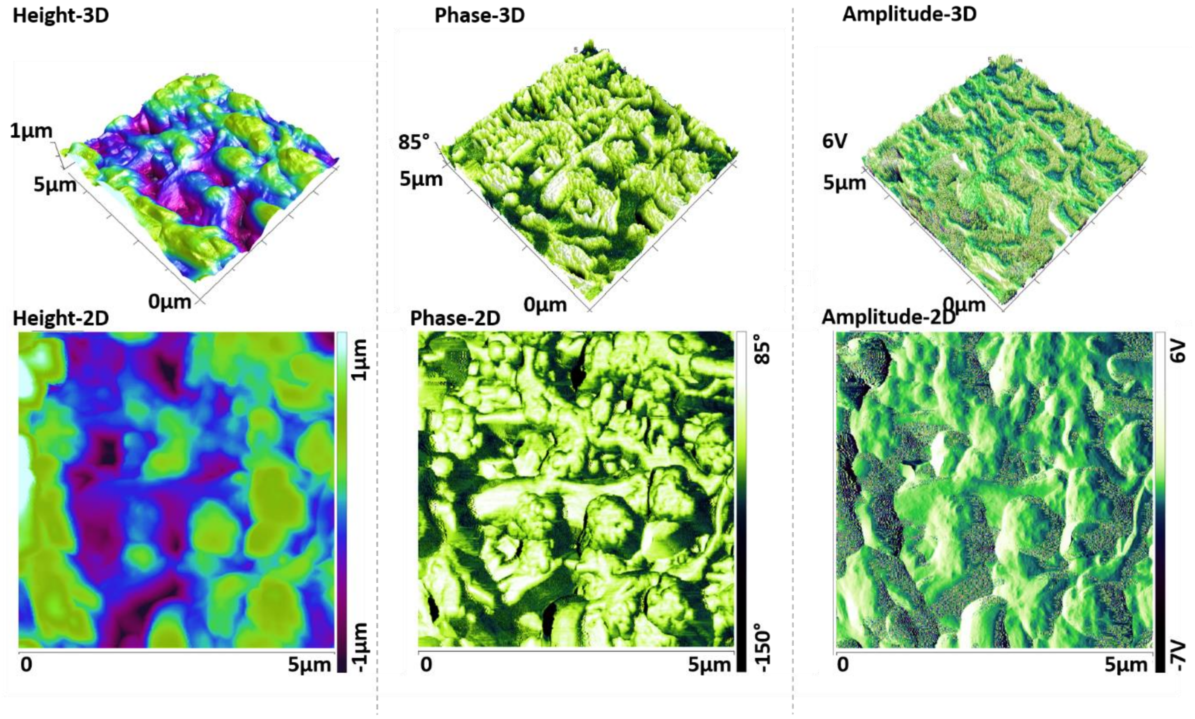

Figure S7. AFM images of hybrid PVDF membrane (P-M-Cl+CS), high, phase and amplitude images in the 2D and 3D projection.

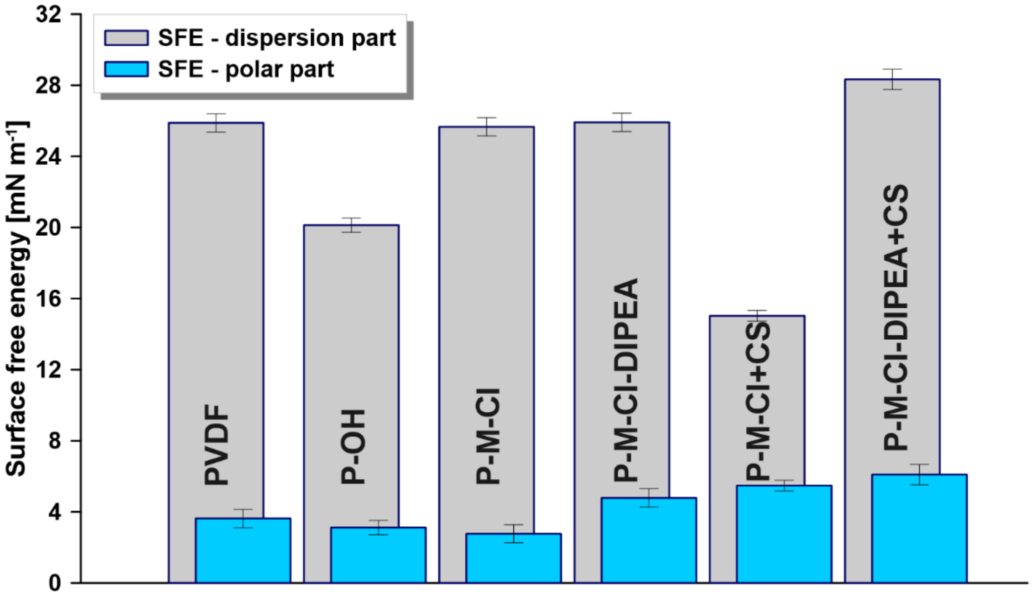

Figure S8. Surface free energy with dispersion and polar components.

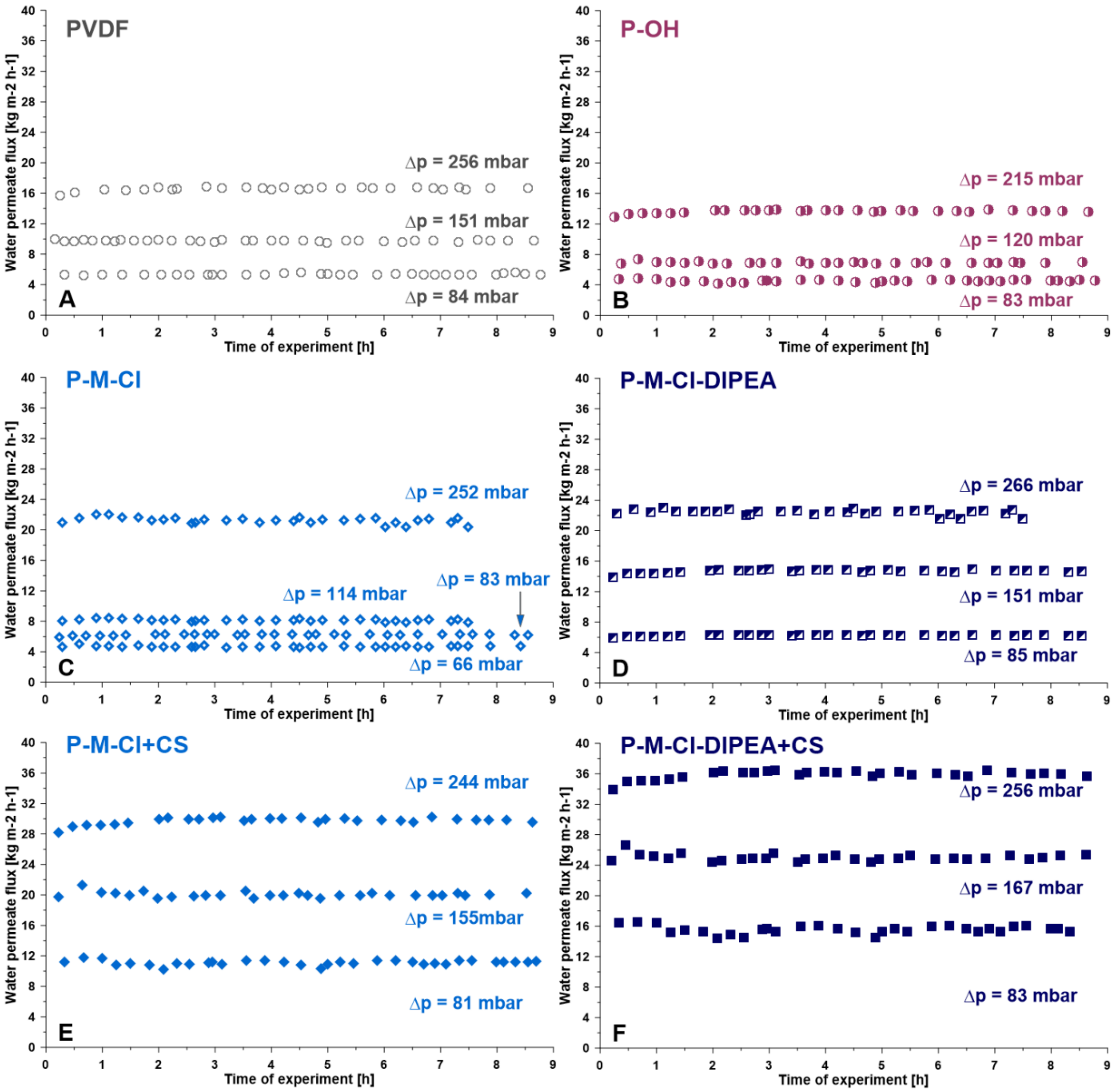

Figure S9. Water flux stability during the AGMD process. A – pristine membrane PVDF, B - activated PVDF (P-OH), C – silanized membrane (P-M-Cl), D – silanized membrane in the presence of catalyst (P-M-Cl-DIPEA), E – hybrid PVDF membrane (P-M-Cl+CS), F – hybrid PVDF membrane prepared in the presence of catalyst (P-M-Cl-DIPEA+CS).

Table S1. Water transport features of the investigated membranes.

Driving force [mbar]	Water flux [kg m^{-2} h^{-1}]	Permeance [kg h^{-1} m^{-2} bar^{-1}]	Overall mass transfer coefficient [kg m^{-2} s^{-1} Pa^{-1}]
		**PVDF**	
84	5.29	102.61	$5.53 \cdot 10^{-6}$
151	9.79	103.08	$3.01 \cdot 10^{-6}$
256	16.34	96.42	$1.58 \cdot 10^{-6}$
		**P-OH**	
83	4.55	90.17	$4.94 \cdot 10^{-6}$
121	6.97	93.48	$3.49 \cdot 10^{-6}$
216	13.43	96.02	$1.91 \cdot 10^{-6}$
		**P-M-Cl**	
65	4.70	117.15	$8.11 \cdot 10^{-6}$
83	6.20	121.12	$6.57 \cdot 10^{-6}$
114	8.15	115.64	$4.56 \cdot 10^{-6}$
252	18.66	111.98	$1.87 \cdot 10^{-6}$
		**P-M-Cl-DIPEA**	
85.00	8.55	163.10	$8.64 \cdot 10^{-6}$
151.26	14.66	153.00	$4.44 \cdot 10^{-6}$
266.00	22.43	126.74	$1.99 \cdot 10^{-6}$
		**P-M-Cl+CS**	
81.00	11.05	221.83	$12.40 \cdot 10^{-6}$
155.00	20.03	204.20	$5.79 \cdot 10^{-6}$
244.00	29.78	185.90	$3.22 \cdot 10^{-6}$
		**P-M-Cl-DIPEA+CS**	
83.40	15.55	304.42	$16.61 \cdot 10^{-6}$
167.00	25.00	236.46	$6.21 \cdot 10^{-6}$
256.00	35.88	211.40	$3.46 \cdot 10^{-6}$
